# Characterization of immunomodulating agents from *Staphylococcus aureus* for priming immunotherapy in triple-negative breast cancers

**DOI:** 10.1038/s41598-024-51361-8

**Published:** 2024-01-08

**Authors:** Chin-Chih Liu, Matthew Wolf, Ruth Ortego, Dennis Grencewicz, Tammy Sadler, Charis Eng

**Affiliations:** 1 Cleveland Clinic, Genomic Medicine Institute, Lerner Research Institute, 9500 Euclid Avenue NE50, Cleveland, OH 44195 USA; 2 Department of Molecular Medicine, Cleveland Clinic Lerner College of Medicine, Case Western Reserve University, Cleveland, OH 44195 USA; 3 Cleveland Clinic, Center for Personalized Genetic Healthcare, Medical Specialties Institute, Cleveland, OH 44195 USA; 4 Cleveland Clinic, Taussig Cancer Institute, Cleveland, OH 44195 USA; 5 Department of Genetics and Genome Sciences, Case Western Reserve University School of Medicine, Cleveland, OH 44106 USA; 6 Germline High Risk Cancer Focus Group, Case Comprehensive Cancer Center, Case Western Reserve University, Cleveland, OH 44106 USA

**Keywords:** Bacterial toxins, Breast cancer

## Abstract

Immunotherapy, specifically immune checkpoint blockade (ICB), has revolutionized the treatment paradigm of triple-negative breast cancers (TNBCs). However, a subset of TNBCs devoid of tumor-infiltrating T cells (TILs) or PD-L1 expression generally has a poor response to immunotherapy. In this study, we aimed to sensitize TNBCs to ICB by harnessing the immunomodulating potential of *S. aureus*, a breast-resident bacterium. We show that intratumoral injection of spent culture media from *S. aureus* recruits TILs and suppresses tumor growth in a preclinical TNBC model. We further demonstrate that α-hemolysin (HLA), an *S. aureus*-produced molecule, increases the levels of CD8^+^ T cells and PD-L1 expression in tumors, delays tumor growth, and triggers tumor necrosis. Mechanistically, while tumor cells treated with HLA display Gasdermin E (GSDME) cleavage and a cellular phenotype resembling pyroptosis, splenic T cells incubated with HLA lead to selective expansion of CD8^+^ T cells. Notably, intratumoral HLA injection prior to ICB augments the therapeutic efficacy compared to ICB alone. This study uncovers novel immunomodulatory properties of HLA and suggests that intratumoral administration of HLA could be a potential priming strategy to expand the population of TNBC patients who may respond to ICB.

## Introduction

Breast cancer is the most common cancer worldwide and the second leading cause of cancer-related deaths in women in the United States (US)^1^ The survival rate of BC varies depending on the extent of disease at the time of initial diagnosis. As such, outcomes are dependent on the stage of disease and breast cancer subtype. Triple-negative breast cancer (TNBC) is an especially aggressive subtype characterized by the lack of expression of estrogen receptor (ER), progesterone receptor (PR), and human epidermal growth factor receptor 2 (HER2/ERBB2)^2^. Stage for stage, TNBCs have the least favorable prognosis due to the paucity of actionable therapeutic targets and the tendency to develop resistance to chemotherapy with early recurrence. Thus, most proponents would prefer strategies addressing the early stages of TNBC.

The immune-checkpoint inhibitors (ICIs) targeting programmed death 1 (PD-1)/programmed death ligand 1 (PD-L1) pathway have been used to treat selected cases of breast cancers according to the benefits found in clinical trials^3,4^ Agents which are used in this setting include pembrolizumab for metastatic and early-stage, high-risk TNBC^5,6^, and dostarlimab for advanced tumors of any type, including breast cancers, with DNA mismatch repair deficiency (dMMR)^7^. Before immunotherapy becomes a promising treatment strategy for breast cancers in general, there are many challenges including low response rate^8^, resistance to immune therapy^9,^ and immune-related adverse effects^10^. Only a subset of TNBC patients with a pre-existing “hot” (inflamed) tumor immune microenvironment (TIME) that contains high PD-L1 expression or enriched tumor-infiltrating lymphocytes (TILs) may benefit from the combined treatment of chemotherapy and pembrolizumab^11,12^. For TNBC patients with fewer TILs or low PD-L1 expression, there is an unmet need to develop immune priming approaches to recruit TILs, which could sensitize TNBCs to both immunotherapy and chemotherapy^13^.

The gut microbiome may influence the responsiveness to immunotherapy as well. Studies have demonstrated that among cancer patients who underwent immunotherapy, the composition of the gut microbiome varies between responders and non-responders^14–16^. Furthermore, better immunotherapy outcomes were observed in experimental mice that received fecal transplantation from responder than non-responder cancer patients^14–16^. In addition to the gut, recent studies have identified microbiota in solid tumors^17,18^. Analyses of clinical samples suggested that these tumoral microbes may affect the TIME of the cancer types with high microbial biomass such as oral and colorectal cancers^18^. Nevertheless, for cancers with low microbial biomass such as breast cancers, the function of the tumoral microbiome is still not fully understood. A recent murine study proposed that the intracellular microbiota residing in mammary tumors could promote metastasis^19^. However, it remains elusive whether breast tumoral microbiota could functionally influence carcinogenesis, TIME, and treatment responsiveness in a taxa-specific manner.

We have previously identified differences in the human breast microbiome between malignant (tumoral) and non-malignant (normal) breast tissues^20^. Our study found that the microbial community in breast tumors is less diverse than that in normal breast tissues. Many bacterial genera that are prevalent in normal breast tissues were found depleted in breast tumors, including *Staphylococcus*. We also found that *Staphylococcus* positively correlates with the degree of infiltrating T cells in tumor-adjacent normal breast tissues. Furthermore, our 16S rRNA gene sequencing identified the reads that specifically represent *Staphylococcus aureus* (*S. aureus*) in both human non-malignant breast tissues and breast tumors. *S. aureus* has been reported with potential immune-stimulating and/or tumor-suppressive functions. For example, a preclinical study found intratumoral injections of *S. aureus* inhibit glioblastoma tumor growth via modulating TIME^21^. There are also clinical cases where GBM patients with intracranial *S. aureus* infection displayed longer survival^22^. These findings suggest *S. aureus* may produce molecules with activity in stimulating anti-tumor immunity.

In this study, we found that the spent media from *S. aureus* cultures when injected intratumorally could increase TILs and interrupt tumor growth in a preclinical model of TNBC. Among the *S. aureus*-produced immunomodulating compounds, we identified α-hemolysin (HLA) as a potentially functional molecule in the *S. aureus* spent media that could inflame TIME by inducing the infiltration of CD8^+^ cytotoxic T cells and PD-L1 expression, accompanied by the inhibition of tumor growth and robust induction of tumor necrosis of murine mammary tumors. Our in vitro experiments further uncovered divergent influences of HLA on tumor cells and T cells. On the one hand, HLA-treated tumor cells displayed the cleavage of Caspase-3 and Gasdermin E (GSDME), a pyroptosis-like cellular morphology, and cell lysis, but on the other hand, HLA-treated splenic T cells showed selective expansion of CD8^+^ T cells. More importantly, by leveraging the EO771 TNBC model, we found that prior immune priming by intratumoral HLA injections augments the therapeutic efficacy of the anti-PD-1 antibody and extends the survival of tumor-bearing mice. Our observations uncover the activities of *S. aureus*-HLA in increasing TILs and transforming the TIME, which could potentially boost the effects of immunotherapy when administered intratumorally.

## Results

### Intratumoral administration of *S. aureus* spent media increases tumor-infiltrating T cells and impedes tumor growth

**Figure 1:**
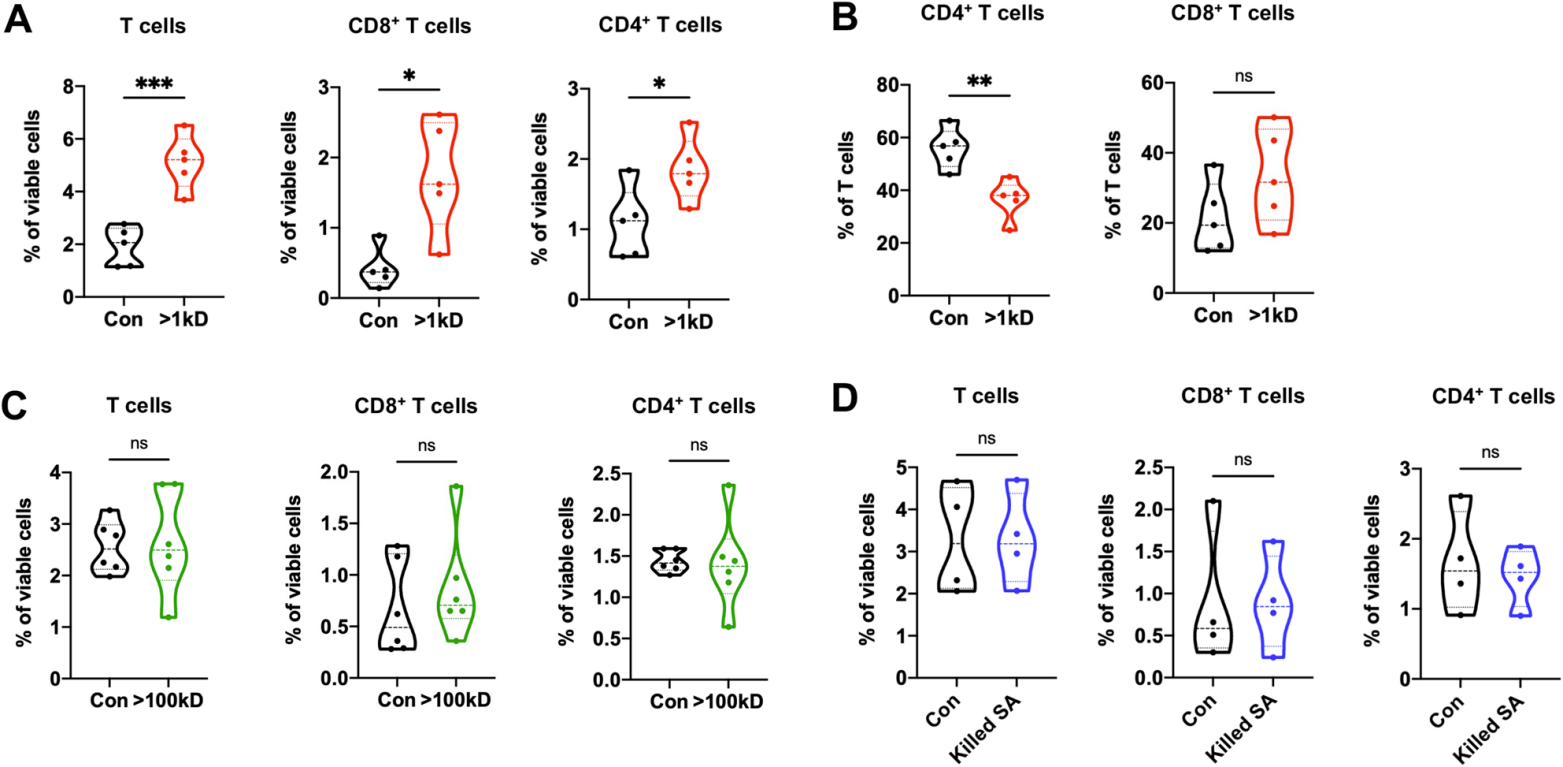
Immunomodulating effects of *S. aureus* derivatives on tumor-infiltrating lymphocytes in TNBC. (**A**) and (**B**), 4T1 tumors were injected with > 1 kD molecules from *S. aureus* spent media (> 1 kD, in red, n = 5) or control media (Con, in black, n = 5). Flow cytometric analysis was performed to determine the percentage of total CD3^+^ T cells, CD8^+^ T cells, and CD4^+^ T cells among viable cells (**A**) and CD4^+^ T cells and CD8^+^ T cells among total T cells (**B**). (**C**) and (**D**), 4T1 tumors were injected with > 100 kD molecules from *S. aureus* spent media (> 100 kD, in green, n = 6) or control media (Con, in black, n = 6) (**C**), and heat-killed *S. aureus* (killed SA, in blue, n = 4) or control DPBS (Con, in black, n = 4) (**D**). The percentage of total T cells, CD8^+^ T cells, and CD4^+^ T cells among viable cells were determined for each treatment ((**C**) and (**D**)). Data are presented as median with quartiles (truncated violin plots). Unpaired two-tailed Student’s t-test. *P < 0.05; **P < 0.01; ***P < 0.001; *ns,* not significant.

The tumor-suppressive functions of *S. aureus* from previous studies prompted us to investigate whether *S. aureus* secretes molecules that could increase T cell infiltration in breast tumors^21,22^. To this end, we assessed the immunomodulatory activity of *S. aureus*’ spent culture media as well as heat-killed *S. aureus*. We found that injections of > 1 kilodaltons (kD) molecules from *S. aureus* spent media into 4T1 tumors increased the fraction of total CD3^+^ T cells and both CD8^+^ and CD4^+^ T cells in the tumors compared to the control treatment (Fig. 1A). Moreover, injections of > 1 kD molecules affected the composition of T cells where CD8^+^ populations displayed an increasing trend and CD4^+^ populations were significantly reduced (Fig. 1B). However, no increase in T cell fraction was observed in the tumors injected with *S. aureus* spent media containing > 100 kD molecules nor heat-killed *S. aureus* (Fig. 1C,D), even though both displayed other influences on T cell activation and innate immune populations (Supplementary Fig. 1A,B). Furthermore, > 1 kD molecules from *S. aureus* spent media increased the ratio of M1-like (anti-tumor phenotype, MHC-II^high^, CD206^−^) to M2-like (pro-tumor phenotype, MHC-II^low^, CD206^+^) macrophages (M1/M2) (Supplementary Fig. 1C)^23^. These data suggest that there may be *S. aureus*-derived molecules in the spent media with a 1–100 kD range of molecular weight that could increase TILs when administered intratumorally.

**Figure 2:**
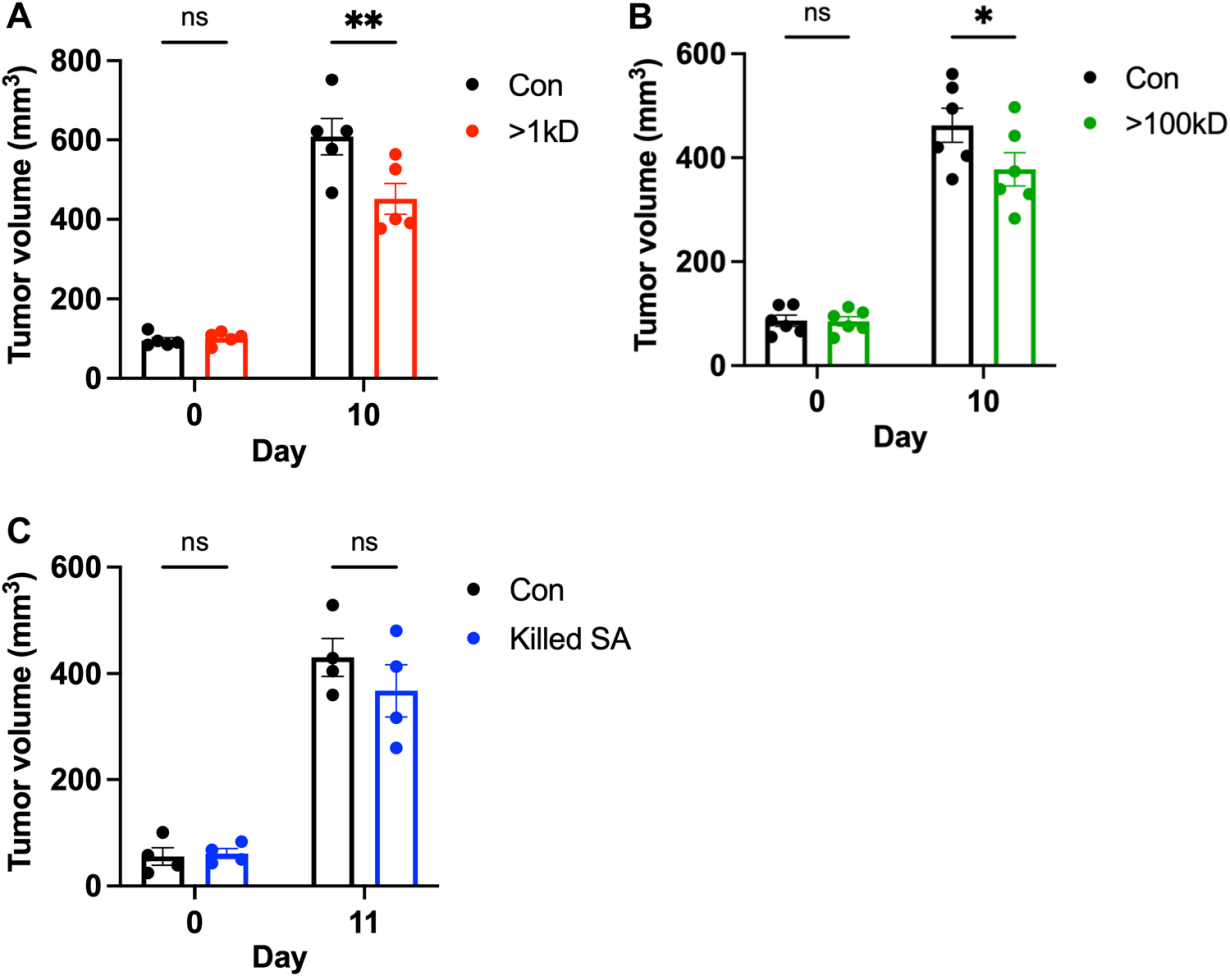
Interrogation of tumor-suppressive effects of *S. aureus* derivatives on tumor growth. (**A**–**C**), The volumes of 4T1 tumors that were injected with > 1 kD molecules from *S. aureus* spent media (> 1 kD, in red, n = 5) or control media (Con, in black, n = 5) (**A**), > 100 kD molecules from *S. aureus* spent media (> 100 kD, in green, n = 6) or control media (Con, in black, n = 6) (**B**), and heat-killed *S. aureus* (Killed SA, in blue, n = 4) or control DPBS (Con, in black, n = 4) (**C**). The time of tumor measurement is indicated as the days after the initial injection. Data are presented as mean ± s.e.m. Two-way analysis of variance (ANOVA) with multiple comparisons. *P < 0.05; **P < 0.01; *ns,* not significant.

In addition to immunomodulatory effects, intratumoral injections of *S. aureus* spent media containing > 1 kD or > 100 kD molecules significantly reduced 4T1 tumor volumes (Fig. 2A,B). However, injections of heat-killed *S. aureus* did not cause significant changes in tumor sizes (Fig. 2C). These results suggest that *S. aureus* spent media contain tumor-suppressive molecules that could antagonize tumor growth.

### α-hemolysin administration increases CD8^+^ T cells and PD-L1 expression in tumors

**Figure 3:**
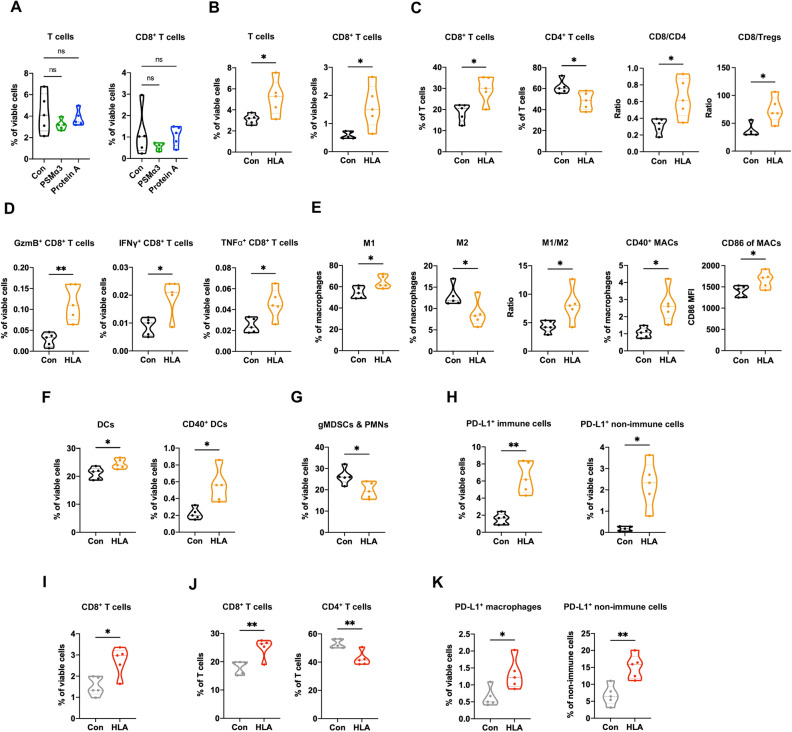
Characterization of immunomodulating effects of *S. aureus* molecules on tumor immune microenvironment. (**A**) Six days after 4T1 tumors were injected with 20 μg of PSM⍺3 (in green, n = 5), 4 μg of Protein A (in blue, n = 5), or vehicle control (Con, in black, n = 5), flow cytometric analysis was performed to determine total T cells and CD8^+^ T cells in tumors. (**B**–**H**), Six days after 4T1 tumors were injected with 1 μg of HLA (in orange, n = 5) or vehicle control (in black, n = 5), flow cytometric analysis was performed to enumerate the immune cells in tumors, which includes the percentage of total T cells and CD8^+^ T cells among viable cells (**B**), the percentage of CD8^+^ and CD4^+^ T cells among total T cells as well as CD8/CD4 and CD8/regulatory T cells (Tregs) ratios (**C**), the percentage of GzmB^+^ , TNFα^+^ , and IFNγ^+^ CD8 T cells among viable cells (**D**), the percentage of M1-like (M1, CD206^-^ MHC-II^high^), M2-like (M2, CD206^+^ MHC-II^low^), CD40^+^ macrophages (MACs) among macrophages, M1/M2 ratio, and CD86 median fluorescence intensity (MFI) of macrophages (**E**), the percentage of total dendritic cells (DCs) and CD40^+^ DCs among viable cells (**F**), the percentage of gMDSCs and PMNs among viable cells (**G**), and the percentage of PD-L1^+^ immune and nonimmune cells among viable cells (**H**). (**I**–**K)**, Six days after the EO771 tumors were injected with 2 μg of HLA (in red, n = 5) or vehicle control (in gray, n = 5), flow cytometric analysis was performed to determine the immune cells in tumors, which includes the percentage of CD8^+^ T cells among viable cells (**I**), the percentage of CD8^+^ and CD4^+^ T cells among total T cells (**J**), and the percentage of PD-L1^+^ macrophages among viable cells and PD-L1^+^ non-immune cells among non-immune cells (**K**). Data are presented as median with quartiles (truncated violin plots). Unpaired two-tailed Student’s t-test. *P < 0.05; **P < 0.01; *ns,* not significant.

To identify if there are known immunomodulatory molecules produced by *S. aureus* that could recapitulate the immune-stimulating effects of *S. aureus* spent media on TILs (Fig. 1A), we first focused on Phenol-soluble modulin alpha 3 (PSM⍺3) and Protein A. These two *S. aureus* molecules fall within 1–100 kD and are known to induce inflammatory responses via recruiting leukocytes, especially polymorphonuclear leucocytes (PMNs)^24,25^. Consistent with their known activity, our flow cytometric analysis revealed that six days following their injections into 4T1 tumors, both PSM⍺3 and Protein A showed a trend of increasing CD11b^+^ Ly6G^+^ Ly6C^low^ cells, which represent PMNs and granulocytic myeloid-derived suppressor cells (gMDSCs) (Supplementary Fig. 2A). However, neither of these two molecules affected the abundance of total and CD8^+^ T cells in 4T1 tumors (Fig. 3A). Next, we turned our attention to HLA, a 33 kD *S. aureus*-secreted molecule that can activate inflammasomes^26^, protein complexes known to initiate inflammatory responses. Flow cytometric analysis showed that six days after the intratumoral injections, HLA led to a significant upregulation of total T cells and CD8^+^ T cells in 4T1 tumors (Fig. 3B), which is similar to the effects observed with > 1 kD *S. aureus* supernatant (Fig. 1A,B). HLA also increased the ratios of CD8^+^ to CD4^+^ and CD8^+^ to regulatory T cells (Tregs, an immunosuppressive subset of CD4^+^ T cells) and stimulated CD8^+^ T cells to produce granzyme B (GzmB), interferon gamma (IFNγ), and tumor necrosis factor alpha (TNFα) (Fig. 3C,D). The Tregs and the T cell populations that express T cell exhaustion marker PD-1 were not altered by HLA (Supplementary Fig. 3A,B). In addition to T cells, HLA increased the ratio of M1-like to M2-like macrophages (M1/M2) (Fig. 3E). Concomitantly, HLA augmented the expression of other M1-associated markers including CD40 and CD86 (Fig. 3E), which further supports that HLA shifted macrophages to anti-tumor phenotypes. Besides macrophages, HLA administration led to increased fractions of total and CD40^+^ dendritic cells (DCs) and a reduction of gMDSCs and PMNs (Fig. 3F,G). Overall, these holistic alterations of adaptive and innate immune cells are indicative of enhanced anti-tumor immune responses and were accompanied by the induction of PD-L1 expression in both tumor cells and immune cells (Fig. 3H). The activities of HLA in recruiting CD8^+^ T cells and inducing PD-L1 expression were additionally validated in EO771 tumors, another syngeneic mouse model for TNBCs (Fig. 3I–K)^27,28^.

We also investigated how intratumoral administration of HLA influences the tumor-draining lymph nodes (dLNs). Similar observations in the tumors (Fig. 3B) were also seen in the dLNs of the 4T1 model whereby HLA injection increased CD8^+^ T cells (Supplementary Fig. 4A). In the dLNs of EO771 model, HLA altered the T cell composition by increasing CD8^+^ and decreasing CD4^+^ T cell populations among total T cells (Supplementary Fig. 4B,C), which are also consistent with the changes observed in tumors (Fig. 3J). To investigate the acute effects of HLA on dLNs, within 24 h after the second HLA injection, dLNs from EO771 tumors were collected and subjected to flow cytometric analysis. The data showed that HLA increases DCs, GzmB^+^ CD4^+^ T cells, and the ratio of CD8^+^ to Tregs in dLNs (Supplementary Fig. 4D–F). Taken together, these results suggest that HLA could increase CD8^+^ T cells and PD-L1 expression in both 4T1 and EO771 tumors as well as influence innate immune cells to form a TIME favoring anti-tumor immune responses.

### α-hemolysin treatment restricts tumor growth and induces tumor necrosis

**Figure 4:**
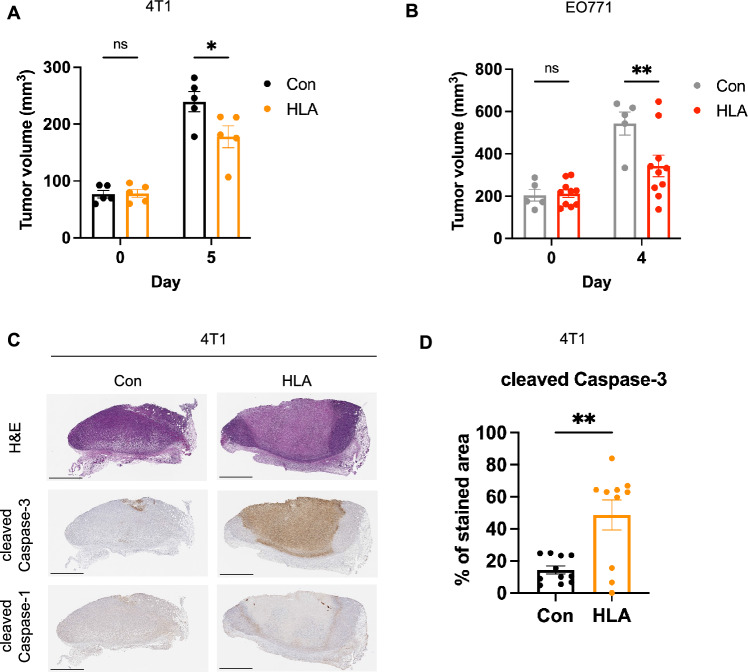
⍺-hemolysin impedes tumor growth and induces tumor necrosis. (**A**) The volumes of 4T1 tumors before (day 0) and after (day 5) intratumoral injections with 1 μg of HLA (in orange, n = 5) or vehicle control (Con, in black, n = 5). (**B**) The volumes of EO771 tumors before (day 0) and after (day 4) intratumoral injections with 2 μg of HLA (in red, n = 10) or vehicle control (in gray, n = 5). (**C**) Representative images of 4T1 tumors that were isolated 2 days after the intratumoral injection of 1 μg of HLA or vehicle control (Con) and subjected to H&E stain (upper panel) and immunohistochemistry (IHC) staining for cleaved caspase-3 (middle panel) and cleaved caspase-1 (lower panel). Scale bars are shown in black at 1000 μm. (**D**) Quantification of the tumoral area that were stained with the cleaved caspase-3 antibody in different treatment groups based on the IHC staining of (**C**) (n = 11 or 10). Data are presented as mean ± s.e.m ((**A**), (**B**), and (**D**)). Two-way analysis of variance (ANOVA) with multiple comparisons ((**A**) and (**B**)). Unpaired two-tailed Student’s t-test (**D**). *P < 0.05; **P < 0.01; *ns*, not significant.

We found that intratumoral injection of HLA significantly reduced tumor size in 4T1 and EO771 models (Fig. 4A,B). This tumor-suppressive effect was not found with PSM⍺3 or Protein A treatment (Supplementary Fig. 2B). Furthermore, HLA-injected 4T1 tumors displayed massive tumor necrosis with a significantly stronger expression of cleaved Caspase-3 when compared to vehicle-treated tumors (Fig. 4C,D). Interestingly, HLA-induced tumor necrotic regions did not express cleaved Caspase-1 (Fig. 4C).

### α-hemolysin induces tumor cell lysis in vitro and stimulates the cleavage of Caspase-3 and GSDME

**Figure 5:**
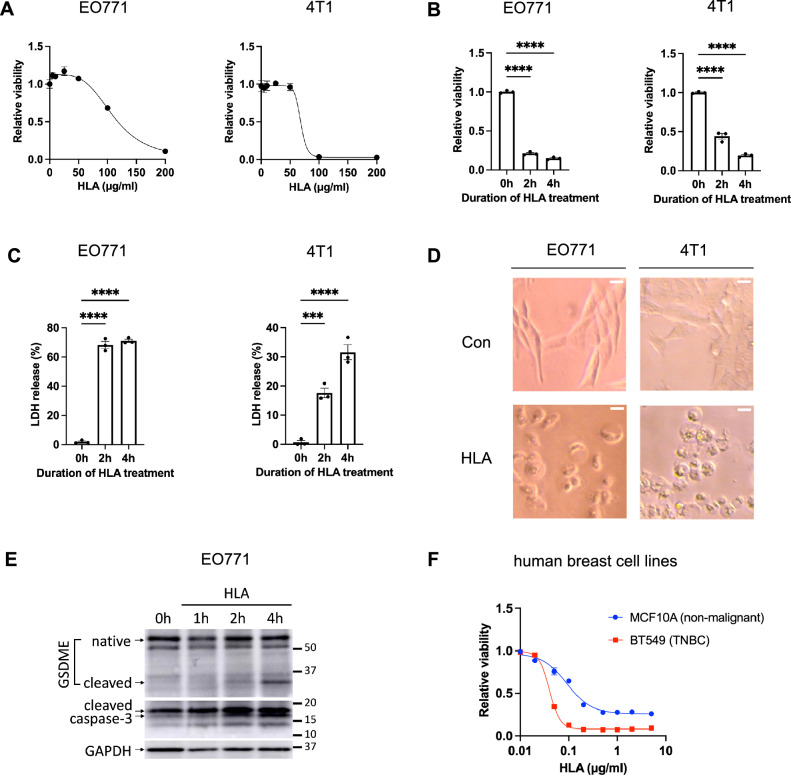
⍺-hemolysin induces tumor cell lysis in vitro and stimulates the cleavages of Caspase-3 and GSDME. (**A**) The dose–response curve of the viability of EO771 (left panel) and 4T1 tumor cells (right panel) that were treated with 5 to 200 μg/ml of HLA for 18 h (n = 3). (**B**) The viability of EO771 (left panel) and 4T1 cells (right panel) that were treated for 0, 2, and 4 h with 200 μg/ml and 100 μg/ml of HLA, respectively. (**C**) The levels of lytic cell death were measured by the percentage of LDH release in culture media of EO771 (left panel) and 4T1 cells (right panel) that were treated for 0, 2, and 4 h with 200 μg/ml and 100 μg/ml of HLA, respectively. (**D**) The representative bright-field microscopic images of EO771 cells treated with 200 μg/ml of HLA or vehicle control (Con) for 90 min (left) and 4T1 cells treated with 100 μg/ml of HLA or vehicle control (Con) for 2 h (right). Scale bars are shown in white at 20 μm. (**E**) EO771 cells were treated with 200 μg/ml of HLA for 0, 1, 2, and 4 h and the protein expression of cleaved GSDME and cleaved Caspase-3 were analyzed by Western Blot. Original blots are presented in Supplementary Information Source data 1. (**F**) The dose–response curve of the viability of MCF10A (in blue) and BT549 cells (in red) that were treated with 0.01 to 5 μg/ml of HLA for 18 h (n = 3 for 0.01–2 μg/ml, n = 2 for 5 μg/ml). Data are presented as mean ± s.e.m. One-way analysis of variance (ANOVA) with multiple comparisons ((**B**) and (**C**)). ***P < 0.001; ****P < 0.0001.

The profound tumor necrosis induced by HLA prompted us to test its tumoricidal effects in vitro. We treated murine TNBC cells with different concentrations of HLA and found that treatment with 200 μg/ml and 100 μg/ml of HLA reduced > 90% of the viability of EO771 and 4T1 cells, respectively (Fig. 5A). With these doses, HLA treatment notably reduced cell viability by inducing acute cell lysis within 2 h of treatment (Fig. 5B,C). Microscopic images of HLA-treated cells showed a swollen and balloon-like cell morphology (Fig. 5D), which together with cell lysis are hallmarks of pyroptosis^29–31^, a form of immunogenic cell death mediated by pore-forming Gasdermin protein family^32^. Accordingly, we gauged the expression of pyroptosis pathways by immunoblotting. We identified cleaved Caspase-3 and cleaved Gasdermin E (GSDME), but not cleaved Caspase-1 or cleaved Gasdermin D (GSDMD), were upregulated at 2 h of HLA treatment in EO771 cells (Fig. 5E, Supplementary Fig. 5). This selective cleavage of Caspase-3 induced by HLA treatment is consistent with the phenotype observed in HLA-treated tumors in vivo (Fig. 4C,D). However, HLA-induced lytic cell death could not be blocked by the inhibitor of Caspase-3 (Z-DEVD-FMK), pan-Caspase (Z-VAD-FMK), Caspase-1/4 (VX-765), NLRP3 inflammasome (MCC950), necroptosis (Necrostatin-1), and ferroptosis (ferrostatin-1) (Supplementary Fig. 6A–D). These results implicate that HLA, as a pore-forming protein^33,34^, may directly lyse tumor cells by forming pores on the cell membranes, thereby independent of cell death pathways. To evaluate the translational potential of HLA in the treatment of human TNBC, we assessed the tumoricidal activity of HLA against BT549, a human TNBC cell line, and MCF10A, a normal non-malignant human breast epithelial cell line. We found the LC50 against BT549 and MCF10A is approximately 0.04 and 0.15 μg/ml, respectively, suggesting BT549 is more sensitive to HLA treatment than MCF10A (Fig. 5F). Moreover, the LC50 against BT549 is less than 1/1000 of that against murine TNBC cell lines (Fig. 5A), indicating HLA possesses more potent tumoricidal activity against human TNBC than murine TNBC cells.

### α-hemolysin differentially affects CD8^+^ T cells, CD4^+^ T cells, and Tregs in vitro

**Figure 6:**
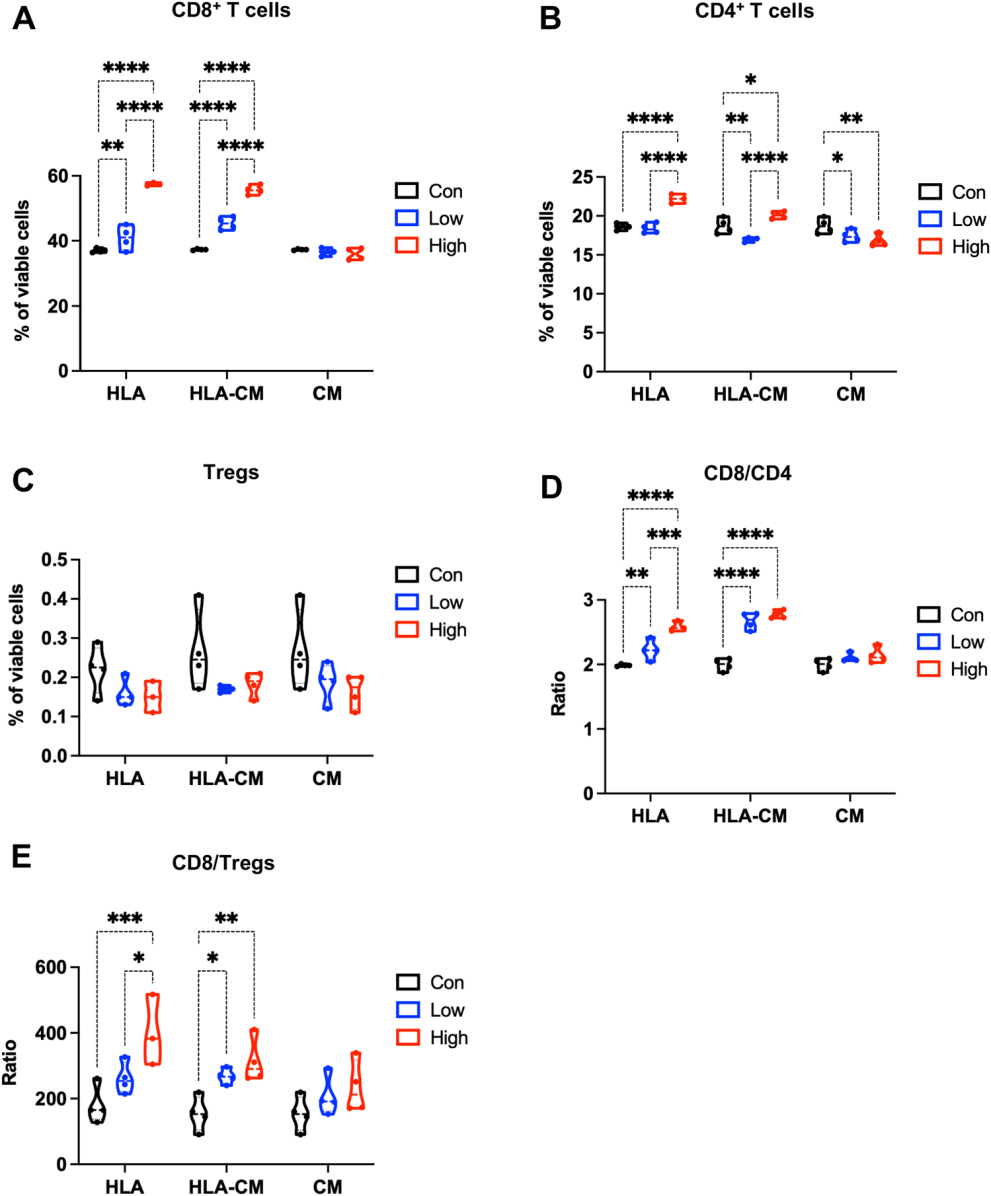
⍺-hemolysin differentially affects CD8 + T cells, CD4 + T cells, and Tregs in vitro. (**A**–**E**) Splenocytes were cultured in the presence of anti-CD3/anti-CD28 activation for 24 h. These in vitro expanded splenic T cells were subjected to a 24 h treatment of either HLA, the conditioned media from the HLA-treated EO771 cells (HLA-CM), or the conditioned media from the vehicle-treated EO771 cells (CM). For HLA treatment, T cells were incubated with 10 μg/ml of HLA (Low, in blue, n = 4), 50 μg/ml of HLA (High, in red, n = 3), and the control vehicle (Con, in black, n = 4). For HLA-CM and CM treatment, T cells were incubated with low and high doses of HLA-CM or CM (in blue and red, respectively, n = 4, referring to the Methods for details). Since HLA-CM and CM treatment groups share the same control group where T cells were treated with EO771 culture media (Con, in black, n = 4), this control group is duplicated in the figures to serve as a control for both HLA-CM and CM groups. Flow cytometric analysis was performed to enumerate the percentage of CD8^+^ cells (**A**), CD4^+^ cells (**B**), and regulatory T cells (Tregs) among viable cells (**C**), as well as CD8^+^/CD4^+^ (**D**) and CD8^+^ /Tregs ratios (**E**). Data are presented as median with quartiles (truncated violin plots). Two-way analysis of variance (ANOVA) with multiple comparisons. *P < 0.05; **P < 0.01; ***P < 0.001; ****P < 0.0001.

We hypothesize that HLA could induce T cells directly or in an indirect manner where the molecules or neoantigens released by HLA-lysed tumor cells stimulate T cells. To identify the direct HLA effects on T cells, we treated the in vitro expanded splenic T cells directly with HLA. To observe the effects on T cells that are mediated by tumor lysis, we incubated splenic T cells with the conditioned media that were collected from the EO771 tumor cells treated with HLA (abbreviated as HLA-CM) at 200 μg/ml for 24 h, the time point when approximately 50 percent of EO771 cells were killed by HLA (Supplementary Fig. 7A). Since longer HLA treatment for 48 and 72 h did not further decrease tumor cell viability (Supplementary Fig. 7A,B), in HLA-CM the cytotoxicity of HLA and possibly the HLA protein itself have already been dampened, which allows us to use HLA-CM to observe the effects attributed to tumor-released molecules instead of HLA itself. Serving as a control for HLA-CM, the conditioned media from vehicle-treated EO771 cells were also collected. Flow cytometric analysis showed that both HLA and HLA-CM treatment strikingly increased the relative abundance of CD8^+^ T cells among viable cells (Fig. 6A). HLA and HLA-CM also slightly expanded CD4^+^ T cell populations among viable cells (Fig. 6B). Neither HLA nor HLA-CM influenced Tregs abundance (Fig. 6C). Intriguingly, HLA and HLA-CM significantly augmented the ratios of CD8^+^ to CD4^+^ and CD8^+^ to Tregs (Fig. 6D,E), which are consistent to HLA’s in vivo activity (Fig. 3C). HLA and HLA-CM did not affect the expression of PD-1 (Supplementary Fig. 8A–D) and did not alter the abundance of CD44^+^ activated T cells (Supplementary Fig. 8E,F). This in vitro enrichment of CD8^+^ T cells by HLA/HLA-CM is similar to the effects of intratumoral HLA administration observed in our mouse models. It revealed that both the HLA protein itself, as well as HLA-mediated tumor lysis, could selectively expand CD8^+^ T cells from T cell populations.

### Prior immune priming by α-hemolysin sensitizes TNBC to immune-checkpoint blockade

**Figure 7:**
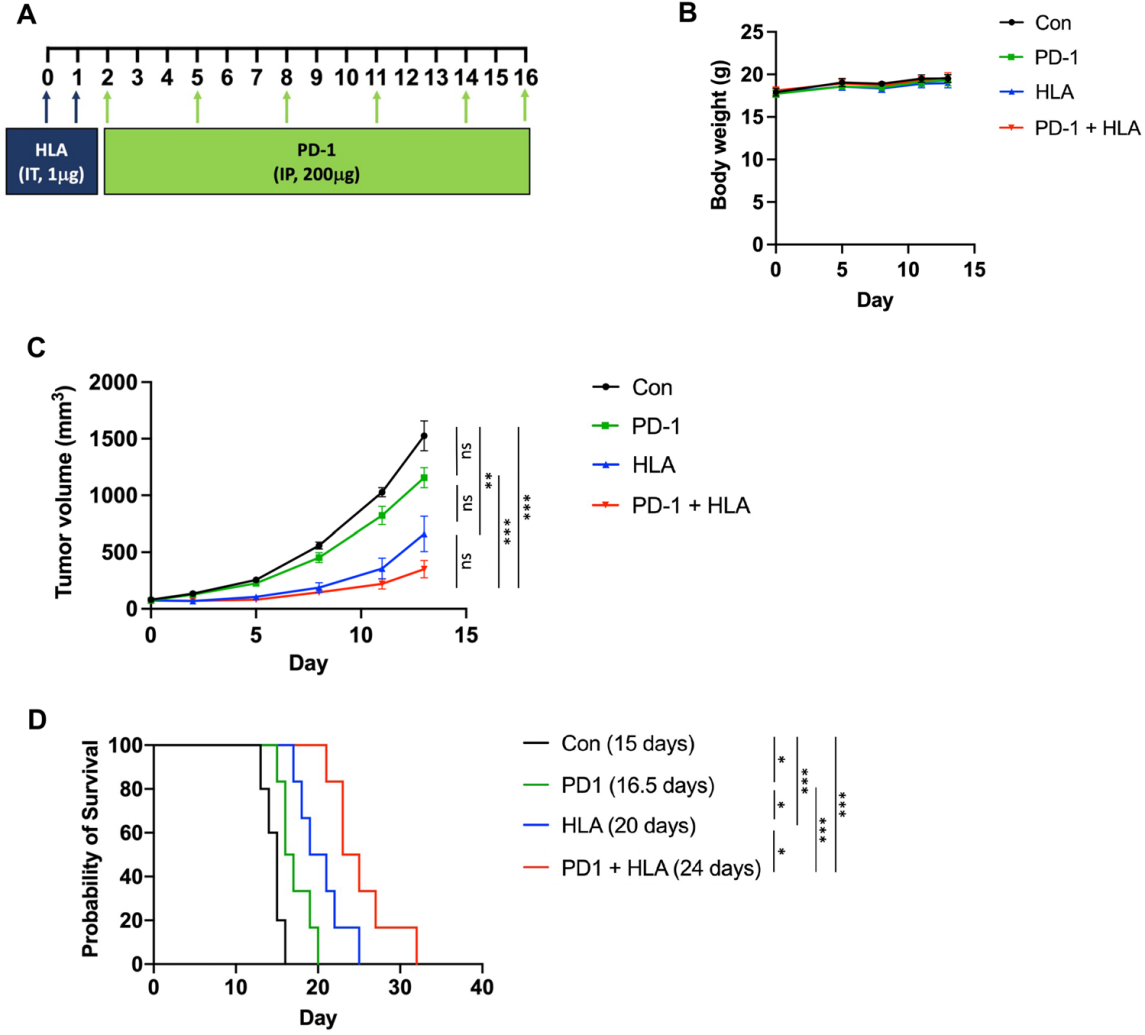
Prior immune priming by ⍺-hemolysin increases the therapeutic efficacy of anti-PD-1 antibody. (**A**) Treatment scheme of (**B**–**D**). HLA and the anti-PD-1 antibody were administered by intratumoral injection (IT) and intraperitoneal injection (IP), respectively. (**B**–**D**) Body weight changes (**B**), tumor growth curves (**C**), and survival curves (**D**) of EO771 tumor-bearing mice subjected to different groups of treatment. The control group was treated with DPBS (IT) and IgG control (IP, 200 μg) (shown in black, n = 5), the HLA group was treated with HLA (IT, 1 μg) and IgG control (IP, 200 μg) (shown in blue, n = 6), the PD-1 group was treated with DPBS (IT) and anti-PD-1 (IP, 200 μg) (shown in green, n = 6), and the PD-1 + HLA group was treated with HLA (IT, 1 μg) and anti-PD-1 (IP, 200 μg) (shown in red, n = 6). Median survivals are shown in parentheses in (**D**). The growth curves of each individual tumors were shown in Supplementary Fig. 7. Data are presented as mean ± s.e.m ((**B**) and (**C**)). Two-way analysis of variance (ANOVA) with multiple comparisons (**C**). Log-rank Mantel-Cox test for survival (**D**). *P < 0.05; **P < 0.01; ***P < 0.001; *ns,* not significant.

The responses of TNBC to neoadjuvant combination therapy of PD-1 blockade and chemotherapy are affected by pre-existing PD-L1 expression and TILs before treatment^11,12,35^. Based on our finding that HLA is a robust inducer of both TILs and PD-L1 expression in TNBCs, we further tested whether prior intratumoral injection of HLA could increase the therapeutic efficacy of systemic anti-PD-1 treatment. We injected HLA into tumors daily for two consecutive days. The day after the second HLA injection, mice were subjected to systemic treatment of either anti-PD-1 or isotype control IgG antibody every second or third day for a total of six treatments (Fig. 7A). These treatment schedules did not result in any adverse effects based on murine appearance, behaviors, and body weight (Fig. 7B). The data showed that anti-PD-1 monotherapy did not significantly affect EO771 tumor growth compared to the control group (Fig. 7C, Supplementary Fig. 9). However, prior priming of HLA before anti-PD-1 treatment (or combination therapy) resulted in strong inhibition of tumor growth compared to both anti-PD-1 monotherapy and control group. While HLA monotherapy led to a significant inhibition of tumor growth, combination treatment displayed more profound effects at a later time point (day 17) reflected by a significant reduction of tumor sizes compared to HLA monotherapy (Supplementary Fig. 10). We also followed the survival of each tumor-bearing mouse until their endpoints were met. Both anti-PD-1 and HLA monotherapies prolonged survival compared to the control group, where HLA provided better survival than anti-PD-1 alone (Fig. 7D). Importantly, the combination treatment of HLA and anti-PD-1 further extended survival when compared to monotherapy of anti-PD-1 or HLA. Taken together, these results suggest that prior priming of anti-tumor immune responses by intratumoral injections of HLA could sensitize TNBC to immune-checkpoint blockade.

## Discussion

TNBC is a heterogeneous disease characterized by varying levels of TILs corresponding to genomic alterations and disease stages^36^. For instance, 41.9% of TNBC patients with heterozygous loss of chromosome 17p (which contains the tumor suppressor *TP53*) demonstrate reduced infiltration of CD8^+^ T cells in tumors^37^. Furthermore, the density of TILs is lower in metastatic TNBC compared to primary tumors^38,39^. Unfortunately, the low density of total and CD8^+^ T cells in TNBC tumors renders both chemotherapy and immunotherapy less effective and is associated with a poorer prognosis^11–13,37^. Clinical studies reported that a short course of neoadjuvant chemotherapy, rather than completing the full course, can boost TIL density and enhance the sensitivity of TNBC to PD-1 blockade^40,41^. Accordingly, unfavorable TIMEs could be improved preemptively by a brief treatment of cytotoxic agents to sensitize TNBC for immunotherapy.

HLA is a virulence factor and a pore-forming toxin secreted by *S. aureus*, a Gram-positive commensal bacterium frequently identified in human nares, skin, and breast. Due to its activity in perforating the cell membrane, HLA secreted by engineered bacteria displayed in vitro cytotoxicity against cancer cell lines, which had once made HLA a potential therapeutic payload for bacteria-based cancer therapy^42–44^. Despite HLA could kill cancer cells in culture dishes, treating tumor-bearing mice with bacteria-based HLA delivery failed to provide survival benefits since this treatment was unbearable and accompanied by profound toxicity (including liver damage), which limits its further development for cancer therapy^42^. To overcome this obstacle, a safer approach for HLA delivery is needed to be developed and validated in preclinical cancer models. Moreover, the lack of knowledge of HLA’s influences on TIME and anti-tumor immunity restrains HLA from being used with cancer immunotherapy.

As an alternative to bacterial drug delivery, intratumoral administration has emerged as an appealing approach in cancer treatment with various ongoing clinical trials^45,46^. This approach is particularly suitable for cytotoxic molecules like HLA since the localized delivery into tumors could potentially decrease systemic toxicity, achieve higher local concentrations, and maximize the release of tumor-associated antigens, thus offering a better therapeutic index and augmenting immune activation^47^. In line with this rationale, in this study, we demonstrated that intratumoral injection of HLA induced extensive tumor necrosis, shaped an inflamed TIME characterized by increased CD8^+^ T cells and PD-L1 expression, and suppressed tumor growth without causing adverse effects in preclinical TNBC models. Furthermore, we found that prior intratumoral injection of immunomodulatory HLA can prime TNBC for response to subsequent anti-PD-1 therapy. Compared to anti-PD-1 treatment alone, the HLA-primed anti-PD-1 therapy significantly inhibited tumor growth and led to prolonged survival of tumor-bearing mice. This strong effect of HLA and anti-PD-1 combined treatment on tumor growth is important since in clinical breast cancer management, the control of primary tumor sizes by neoadjuvant (preoperative) treatment also could reduce the chance of cancer recurrence and lead to a better prognosis^48^. It’s noteworthy that high TIL density has also been associated with improved outcomes of ICI treatment in melanoma, endometrial cancer, colorectal cancer, and non-small cell lung cancer^49^, suggesting the HLA priming therapy has the potential to be applied to other cancer types beyond TNBC to augment the therapeutic efficacy of ICIs.

Here, we demonstrated that intratumoral injection of HLA extensively induced tumor necrotic regions expressing cleaved caspase-3, which was accompanied by increased CD8^+^ T cells within tumors. This suggests that HLA-triggered tumor necrosis is immunogenic. Our in vitro experimental data showed that HLA treatment led to cellular changes resembling pyroptosis, specifically the cleavage of GSDME and a swollen balloon-like cellular morphology. Pyroptosis is a highly immunogenic form of cell death mediated by the Gasdermin protein family^50^, of which GSDME was found with higher expression in ER-negative breast tumors than ER-positive tumors^51^. Mechanistically, in response to pyroptosis stimuli, caspase-3 cleaves GSDME and the released N-terminal fragment of GSDME could form pores from the inner side of the plasma membrane. These pores disrupt the ionic gradients across the cell membrane, increase osmotic pressure, and cause cell swelling, which could subsequently lead to cell rupture, releases of intracellular antigens, and stimulation of immune responses^31,32^. This GSDME-dependent pyroptosis could convert chemotherapy-induced cell death from non-immunogenic apoptosis to immunogenic pyroptosis and augment anti-tumor immune responses in preclinical models^31,52^. GSDME can also stimulate cleavages of Caspase-3 by perforating mitochondrial membranes^53^. These known immune-stimulating functions suggest that GSDME may play a critical role in HLA-induced immune activation.

Surprisingly, even though we observed HLA treatment induced caspase-3 cleavage, HLA-induced cell death was not abrogated by inhibitors targeting caspases, NLRP3 inflammasome, necroptosis, or ferroptosis. It suggests that HLA, as a pore-forming protein, may cause cancer cell death by directly lysing cells without relying on other intracellular molecules^26^. Further studies are required to investigate the specific roles of GSDME and caspase-3 in HLA-induced cell death and immune activation. Furthermore, it is worth noting that approximately 35% of all bacterial toxins are pore-forming proteins^43^. In addition to HLA, it is likely that other lytic molecules produced by bacteria also possess anti-cancer activity and could be explored for intratumoral immunotherapy.

Encouragingly, we found that the LC50 of HLA against BT549, a human TNBC cell line, was less than 1/1000 of that against murine TNBC cell lines including EO771 and 4T1, suggesting HLA may provide an even better anti-cancer activity in TNBC patients than what we have observed in preclinical TNBC models. Moreover, the LC50 of HLA against BT549 is less than 1/3 of that against non-malignant human breast cell line MCF10A, indicating malignant TNBC cells are more vulnerable to HLA treatment than healthy breast cells. Similarly, previous studies also found that human lung cancer cell line A549 is more sensitive to HLA treatment than non-malignant human airway epithelial cell lines like S9 and 16HBE14o-^54^. These differences in HLA sensitivity between cell types and species are possibly attributed to A Disintegrin and Metalloproteinase 10 (ADAM10). ADAM10 is an HLA receptor expressed on the cell membrane, which is required for HLA-binding and HLA-mediated cytotoxicity, especially at low HLA concentrations^55^. Cells with higher surface expression of ADAM10 were found more vulnerable to HLA-triggered cell lysis. For example, human erythrocytes do not express surface ADAM10 and therefore are 16,000 times more resistant to HLA-mediated cytotoxicity than rabbit erythrocytes^55,56^. ADAM10 is also likely involved in breast cancer progression^57,58^. Interestingly, human TNBC cell lines displayed higher levels of ADAM10 expression than human cell lines of ER-positive breast cancer^57^. Consistent results were found in clinical samples where ER-negative breast tumors showed elevated levels of ADAM10 expression than ER-positive tumors^58^. These findings implicate that human TNBC may display superior responsiveness to HLA than other subtypes of human breast cancers due to ADAM10 overexpression. Furthermore, based on the high HLA sensitivity of the human breast cancer cell line BT549 (with an LC50 close to 0.04 μg/ml or 1 nM, Fig. 5F), intravenous (IV) injection is another potential route that could be considered for HLA administration. IV injection or drip has the advantage of delivering a precise dose of HLA quickly in a well-controlled manner, which provides the chance to achieve targeted killing of cancer cells based on the differential HLA sensitivity between malignant and non-malignant breast cells.

Even though HLA displayed robust cytotoxicity against tumor cells, our in vitro experiments using expanded splenic T cells showed that treatment of HLA or the conditioned media from HLA-treated EO771 tumor cells selectively enriched CD8^+^ T cells, which reiterates HLA’s in vivo activity and suggests both the HLA protein itself and the HLA-mediated tumor lysis could increase CD8^+^ T cells to sensitize the tumors to immunotherapy. In addition, HLA’s influences on other cell populations in the tumor microenvironment, such as the reduction of immunosuppressive gMDSCs and PMNs, the increased DCs, and the elevated ratio of M1 to M2 macrophages, may further unleash the anti-tumor activity of CD8^+^ T cells.

Importantly, we cannot exclude the possibility that other *S. aureus*-secreted molecules in the spent media may have contributed to the observed immune activation and tumor suppression (Figs. 1A, 2A). For example, various pore-forming toxins secreted by *S. aureus* include β-hemolysin, Panton-Valentine leukocidins (PVLs), and leucocidin ED/GH may also have the potential to cause cancer cell death^59,60^. Moreover, the *S. aureus*-secreted superantigens, such as Staphylococcal enterotoxin A/B/C (SEA/B/C), have robust activity in T cell expansion and activation, which also possibly triggered immune responses within tumors^61^. Even though there is a possibility that these molecules and HLA may additively or synergistically contribute to the anti-cancer activity of *S. aureus* spent media, here we demonstrate that HLA treatment alone is sufficient to reiterate the effects of *S. aureus* spent media in increasing tumor-infiltrating CD8^+^ T cells and delaying tumor growth.

Our study also sheds light on the potential crosstalk among microbes, cancer cells, and the immune system in breast tissues. Previous preclinical studies have found that bacteria residing in mammary tissues can promote carcinogenesis and metastasis^19,62^. However, clinical studies have reported that antibiotic use is associated with an elevated breast cancer risk^63,64^, worse outcomes of breast cancer treatment^65,66^, and decreased survival among TNBC patients^67^. This implies the existence of potentially tumor-suppressive bacterial taxa in breast tissues. Here, we demonstrated that *S. aureus*-secreted molecules can increase TILs and suppress tumor growth. Accordingly, *S. aureus* may act as a “guard” bacterium in breast tissues where it may antagonize carcinogenesis and cancer progression via eliciting immune surveillance. This hypothesis aligns with the positive correlation between the levels of *Staphylococcus* and T cell density in tumor-adjacent breast tissues and the reduced *Staphylococcus* levels in human breast tumors^20^. Interestingly, systemic decreases of *Staphylococcus* were also observed in the blood of breast cancer patients compared to healthy controls^68^. In addition to *Staphylococcus*, our team and another group consistently found decreased levels of *Streptococcus*, *Lactococcus*, and *Corynebacterium* in human breast tumors compared to healthy breast tissues^20,69^. Intriguingly, *Streptococcus* expresses a toxin that directly cleaves GSDME, which leads to pyroptosis^70^. Therefore, it would be meaningful to investigate the influences of these breast-resident bacteria on various breast cancer phenotypes, TIME, pyroptosis, and treatment responses.

In conclusion, this study highlights the following novel findings. First, we uncover novel immunomodulatory activities of HLA, especially in recruiting CD8^+^ T cells and increasing PD-L1 expression in TIME. Second, we propose intratumoral injection as a safe and effective approach for targeted delivery of HLA into tumors. We demonstrate this method does not cause any toxicities that have been reported before where HLA was delivered by bacteria^42^. Finally, we show that HLA could be incorporated into the modality of TNBC immunotherapy. The combined treatment of HLA and anti-PD-1 antibody has the ability to sensitize TNBC tumors with unfavorable TIMEs to immunotherapy and addresses these tumors at an early stage, paving the way for improved treatment strategies for TNBC patients.

## Methods

### *S. aureus* culture

In order to collect the spent media from *S. aureus* cultures, a single colony of *S. aureus* MN8 (BEI Resources Repository) grown on a Brain Heart Infusion (BHI) agar plate was inoculated into BHI media (Sigma, # 110493) supplemented with menadione (1 mg/l, MP Biomedicals, # 02102259), hematin (1.2 mg/l, Santa Cruz, # sc-207729), histidine (0.2 mM, Tokyo Chemical Industry, # H0149), and L-cysteine hydrochloride (0.5 g/l, J.T.Baker, # 2071-05) and incubated overnight (22 h) at 37 °C in an anaerobic chamber. After a 20 min centrifugation at 3000 *rpm*, the supernatant of the *S. aureus* culture was collected and filtered through a 0.22 μm syringe filter (Sigma, # SLGM33RS). The supernatant was concentrated to a fifth of the original volume (5 times concentrated) using centrifugal filters with 1 kD molecular weight cut-off (MWCO) (Pall, # MAP001C36) or 120 times concentrated via centrifugal filters with 100 kD MWCO (Amicon, # UFC910024). The > 100 kD concentrate was diluted 24 times with DPBS (Dulbecco’s phosphate-buffered saline) to match the concentration of > 1 kD supernatant concentrate. The sterile BHI medium without bacterial inoculation was simultaneously processed in the same way and used for control treatment.

Heat-killed *S. aureus* was obtained by pelleting the overnight anaerobic culture with a 20 min centrifugation at 3000 *rpm*. The *S. aureus* pellet was washed and resuspended in DPBS at a concentration of 5 × 10^9^ CFU (colony-forming units) per ml, which was confirmed by the enumeration on the BHI agar plates. The *S. aureus* suspension was heated to 85 °C for 25 min, with a vigorous vortex every 5 min. The death of *S. aureus* was confirmed by no colony formation on the BHI agar plates.

### Cell culture

4T1 cells were maintained in Roswell Park Memorial Institute (RPMI) medium (Gibco, # 31800-105) supplemented with 10% heat-inactivated fetal bovine serum (FBS) (Gibco, # 10437028) and 100 U/ml Penicillin (Sigma, # P3032-100MU) and Streptomycin (Gibco, # 11860-038). EO771 (# CRL3461) cells were purchased from American Type Culture Collection (ATCC) and were maintained in Dulbecco’s Modified Eagle’s Medium (DMEM) (Caisson Labs, # DMP08-50LT) with 10% heat-inactivated FBS, 20 mM HEPES, and 100 U/ml Penicillin and Streptomycin. EO771 and 4T1 cell lines were confirmed to be without infection of mycoplasma, other bacteria, and viruses by CLEAR (cell line examination and report) PCR Panels detecting rodent and human infectious agents (Charles River). BT549 were maintained in DMEM/F-12 (Caisson Labs, # DFP18) with 10% heat-inactivated FBS, L-glutamine (Caisson Labs, # G010), and 100 U/ml Penicillin and Streptomycin. MCF10A were maintained in Mammary Epithelial Cell Growth Medium (MEGM^™^, Lonza, # CC-3150) without adding GA-1000 and supplemented with 100 ng/ml cholera toxin (Sigma, # C8052). 4T1, BT549, and MCF10A cell lines were authenticated using short tandem repeat DNA profiling (Labcorp).

### Tumor growth and treatment

To develop orthotopic mammary tumors, 4T1 and EO771 cells were trypsinized (Caisson Labs, # T019) and resuspended in DPBS without calcium and magnesium (Cleveland Clinic Lerner Research Institute (CC-LRI) Cell Culture Core). 1 × 10^4^ of 4T1 cells and 5 × 10^5^ to 1 × 10^6^ of EO771 cells were injected into the fourth mammary fat pad of female BALB/c and C57BL/6 J mice (The Jackson Laboratory), respectively, of six to eight weeks old. When the smallest tumor reached at least 30 mm^3^ in volume, mice were randomized with matching tumor volumes in cases and controls before treatment. The day of the first treatment was defined as day 0. To characterize the effects of *S. aureus* derivatives on the TIME and tumor growth, > 1 kD or > 100 kD *S. aureus* spent media was injected into 4T1 tumors on day 0 (with a volume of 70 μl) and day 3, 6, and 10 (with a volume of 100 μl), with corresponding media concentrates without bacterial inoculation serving as control treatments. 20 μl of heat-killed *S. aureus* was injected into 4T1 tumors on day 0 and 4 with DPBS injection serving as the control treatment. To investigate the effects of *S. aureus* molecules on TIME and tumor growth, 20 μg of PSM⍺3 (synthesized by Peptide 2.0 Inc.), 4 μg of Protein A (Sigma, # P6031), 1 μg of HLA (Sigma, # H9395), or DPBS (vehicle control) with a volume of 20 μl was injected into 4T1 tumors on day 0. We observed the effects of HLA on EO771 tumors by injecting 2ug of HLA with a volume of 40 μl into tumors. To interrogate whether immune priming by HLA could improve the therapeutic effects of anti-PD-1 treatment, EO771 tumors were injected with 1 μg of HLA or vehicle control (DPBS) daily for two consecutive days. The day after the second HLA injection, mice were subjected to intraperitoneal injections of anti-PD-1 (Bioxcell, clone RMP1–14, 200 μg) or isotype control IgG (Bioxcell, clone 2A3, 200 μg) every second or third day to complete a total of six treatments according to the treatment scheme (Fig. 7A). Tumor volumes were measured by the digital caliper and calculated by the ellipsoid formula (L × W × W/2). When tumors reached a length of 17 mm in any direction, the mice were euthanized by carbon dioxide (CO_2_) inhalation, which was followed by cervical dislocation to ensure death. The day of euthanasia was used for survival analyses. All protocols of animal experiments were approved by Institutional Animal Care and Use Committee (IACUC) of Cleveland Clinic (protocol number: 00002429). All experimental methods were carried out in accordance with the approved protocol and in accordance with the guidelines and regulations of IACUC. Animal experiments were performed and reported in accordance with Animal Research: Reporting of In Vivo Experiments (ARRIVE) guidelines.

### Immune cell dissociation and flow cytometry

For the profiling of the immune landscape in tumors, 4T1 tumors injected with > 1 kD or > 100 kD *S. aureus* spent media were isolated on day 11. 4T1 tumors injected with heat-killed *S. aureus* were isolated on day 12. 4T1, EO771 tumors, and dLNs were isolated 6 days after the intratumoral injection of HLA, PSM⍺3, or Protein A. Tumors were cut into small pieces and digested with DMEM media supplemented with 1X Collagenase/Hyaluronidase (STEMCELL, # 07912) or 750 μg of Liberase™ TL (Sigma, # 5401020001), 200 μg/ml Deoxyribonuclease I (Sigma, # DN25), < 5% heat-inactivated FBS, 2% penicillin–streptomycin, and 10 mM HEPES at 37 ºC for 30 min with mild agitation. After digestion, cells were passed through 40 μm filters. To obtain cells in dLNs and spleens, lymph nodes and spleens were mashed by syringes on top of 70 μm filters. The dissociated cells from tumors, dLNs, or spleens were resuspended in red blood cell lysis buffer (STEMCELL, # 20120) for 3 to 4 min at room temperature to eradicate red blood cells. Collected cells were stained with viability dye in DPBS (Biotium, # 32018, 1:1000) for 30 min on ice, blocked with anti-mouse CD16/32 antibody (Biolegend, # 156604, 1:100) in staining buffer (DPBS with 0.5% Bovine Serum Albumin (BSA), 2 mM EDTA, and 0.05% NaN_3_) for 20 min on ice. Then the samples were stained with antibodies targeting cell surface immune markers in staining buffer for 30 min on ice, including the antibody specific to CD45 (BD Biosciences, 30-F11, 1:500), CD103 (Thermo Fisher Scientific, 2E7, 1:250), CD49b (Biolegend, DX5, 1:100), CD11b (Thermo Fisher Scientific or BD Biosciences, M1/70, 1:100 or 1:200, respectively), CD86 (Biolegend, GL-1, 1:700), Ly-6C (Biolegend, HK1.4, 1:100), Ly-6G (BD Biosciences, 1A8, 1:100), H-2Kd (BD Biosciences, SF1-1.1, 1:50), I-A/I-E (BD Biosciences, M5/114.15.2, 1:100), PD-L1 (Elabscience, 10F.9G2, 1:100), CD19 (BD Biosciences, 1D3, 1:250), CD3 (Biolegend, 17A2, 1:100), CD11c (BD Biosciences, N418, 1:250), CD206 (Biolegend, C068C2, 1:30), CD4 (BD Biosciences, GK1.5, 1:100), PD-1 (Biolegend, 29F.1A12, 1:500), CD40 (Thermo Fisher Scientific, 1C10, 1:100), F4/80 (Biolegend, BM8, 1:250), CD8a (Biolegend, 53-6.7, 1:500), CD62L (Thermo Fisher Scientific, MEL-14, 1:100), and CD44 (BD Biosciences, IM7, 1:100). Most flow cytometry antibodies used in this study have been serially titrated to identify the optimal doses. After two washes with staining buffer, the cells were resuspended in fixing buffer at room temperature for 60 min and permeabilized by the Perm buffer according to the manufacturer’s instructions (Biolegend, # 424401). Cells were then stained for 45 min at room temperature with antibodies targeting intracellular immune markers, including the antibodies specific to FoxP3 (Biolegend, MF-14, 1:200), Granzyme B (Thermo Fisher Scientific, NGZB, 1:200), IFNγ (Biolegend, XMG1.2, 1:200), and TNFα (Biolegend, MP6-XT22, 1:50). After two washes with Perm buffer, cells were resuspended in staining buffer, filtered through a 35 μm mesh (Corning, # 352235), and analyzed by the spectral cell analyzer SONY ID7000^™^. The resulting FCS data files were then analyzed by FlowJo^™^ Software v10.8.0 (BD Life Sciences) with the gating strategy shown in Supplementary Fig. 11.

### Hematoxylin and eosin (H&E) stain and immunohistochemistry (IHC)

4T1 tumors were injected with 1 μg of HLA or DPBS control in a volume of 20 μl. Two days after the injection, tumors were isolated, fixed in 4% paraformaldehyde, transferred to 70% ethanol, and subsequently embedded in paraffin wax. The 5 µm-thick sections of the paraffin-embedded tumors were mounted on glass slides, deparaffinized with xylene, and rehydrated with a graded ethanol series. H&E staining was completed using a Leica ST5020 Automated Stainer by the Imaging Core of CC-LRI. For IHC, antigen retrieval was performed by incubating sections at 95 °C for 20–25 min in Target Retrieval Solution (Dako, # S169984-2). Sections were incubated with 5% Triton X-100 and 1% H_2_O_2_ for 30 min followed by blocking in 3% normal goat serum (Vector Laboratories, NGS, # S-1000-20) in PBS for at least two hours. The primary antibodies, cleaved caspase-3 (Cell Signaling, # 9664, 1:500) and cleaved caspase-1 (Invitrogen, # PA5-99390, 1:400), were diluted in a blocking solution and incubated at 4 °C overnight. After washed with PBS, the sections were incubated with horseradish peroxidase (HRP)-conjugated secondary antibody (Vector Laboratories, # MP-7451) for 25 min. Sections were washed and developed chromogenically using 3,3’-Diaminobenzidine (DAB) Substrate Kit (Vector Laboratories, # SK-4105). Sections were counterstained with hematoxylin, followed by dehydration in a graded ethanol series, clearing in xylene, and mounting with a toluene-based mounting medium. Images were acquired at 20 × magnification on Leica Aperio AT2 Slide Scanner. Images were analyzed using the open-source digital pathology software, QuPath^71^. To establish relative amounts of cleaved Caspase-3 expression, the function “Detect Positive Staining” was used to determine the percentage of positively stained pixels with optical density (OD) higher than a threshold DAB value of 0.2 OD units.

### In vitro studies

4T1 and EO771 cells were seeded at a density of 5 × 10^3^ cells/well and BT549 and MCF10A cells were seeded at a density of 8 × 10^3^ cells/well into 96-well plates one day prior to the treatment with 0.01-200 μg/ml HLA (Sigma, # H9395; or IBT Bioservices, # 1401-002) for 0–72 h as indicated in the figures and figure legends. To investigate the molecular mechanisms underlying HLA-induced cell death, EO771 and 4T1 cells were pre-treated with 200 μM VX-756 (InvivoGen, # inh-vx765i-1), 30 μM Z-DEVD-FMK (BD Biosciences, # 550378), 30 μM Z-VAD-FMK (BD Biosciences, # 550377), 40 μM MCC950 (InvivoGen, # inh-mcc), 20 μM Necrostatin-1 (Calbiochem, # CAS 4311-88-0), 2 μM Ferrostatin-1 (Sigma, # SML0583), or dimethyl sulfoxide (DMSO) (Sigma, # D2650) as vehicle control for 1.5 h. This was followed by a 2 h co-treatment of HLA (Sigma, # H9395; or IBT Bioservices, # 1401-002) at 200 μg/ml and 100 μg/ml for EO771 and 4T1 cells, respectively, with the continuous incubation of corresponding pre-treatment agents. The viability and lytic cell death were measured by MTS assay (Promega, # G3580) and LDH release assay (Promega, # G1780), respectively, using the microplate reader (BioTek Synergy H1).

Splenocytes were isolated from the spleen of a C57BL/6 J mouse of eight weeks old (The Jackson Laboratory) as described earlier. Splenocytes were seeded into U-bottom 96-well plates at a density of 1.5 × 10^5^ cells/well and cultured with RPMI medium supplemented with 10% heat-inactivated FBS, 100 U/ml Penicillin and Streptomycin, 20 mM HEPES, and 50 μM β-Mercaptoethanol, and activated by 2 μg/ml of anti-CD3 antibody (Bioxcell, clone 2C11) and 1 μg/ml of anti-CD28 antibody (Bioxcell, clone PV-1). After 24 h of activation, the in vitro expanded splenic T cells were treated with 10 or 50 μg/ml of HLA with DPBS treatment serving as vehicle control. Also, splenic T Cells were treated with the conditioned media from HLA-treated and DPBS-treated EO771 cells. To acquire these conditioned media, EO771 cells were seeded into a 6-well plate at a density of 3 × 10^5^ cells/well to grow overnight, which was followed by the treatment with 200 μg/ml of HLA or DPBS for an additional 24 h. The supernatant from the treated EO771 cells was collected as the conditioned media after a centrifuge at 1600 *rpm* and filtered through a 0.22 μm syringe filter. The splenic T cells were treated with these conditioned media at low and high doses where HLA-CM and CM constitute one ninth and one third of the final volume of T cell culture media, respectively. HLA-CM and CM treatment groups share the same control group where T cells were incubated with EO771 culture media. After treatment with either HLA, HLA-CM, or CM for 24 h, cells were collected as single-cell suspension by pipetting and subjected to antibody staining and flow cytometry analysis as described earlier.

### Immunoblotting

EO771 cells were seeded in a 6-well plate and treated with 200 μg/ml of HLA (IBT Bioservices, # 1401-002) for 1, 2, and 4 h. Cell lysates were then harvested by Radioimmunoprecipitation assay (RIPA) buffer (Sigma, # R0278) supplemented with phosphatase inhibitors (Sigma, # P5726, # P0044) and protease inhibitors (Sigma, # P8340). The protein concentration of the lysates was determined by a Bicinchoninic acid (BCA) protein assay (ThermoFisher, # 23227). 10 μg of protein was loaded onto and separated by 4–15% precast polyacrylamide gels (BioRad, # 5671083) and was transferred to a nitrocellulose membrane using the Transblot Turbo apparatus (BioRad, # 1704271). After a one-hour blocking with 5% BSA (Gold Biotechnology, # A-420-500) diluted in Tris-buffered saline with 0.2% Tween^®^ 20 (TBST), membranes were incubated with primary antibodies diluted 1:1000 in blocking buffer overnight at 4 °C. The primary antibodies used include anti-GSDME (Abcam # ab215191), anti-GSDMD (Cell Signaling # 39754), anti-cleaved caspase 3 (Cell Signaling # 9664), anti-cleaved caspase 1 (Cell Signaling # 89332), and anti-GAPDH (Abcam, # ab9484). The secondary antibodies were HRP-conjugated anti-mouse or anti-rabbit IgG (Promega, # W4021 and # W4011). The Western blots were scanned digitally.

### Statistics and reproducibility

Statistical analyses were performed with Prism version 9.0.0 (GraphPad). Unpaired two-tailed Student’s *t*-test and One-way analysis of variance (ANOVA) were used to compare experiments with two and greater than two groups, respectively. Two-way ANOVA with Sidak’s and Tukey’s multiple comparisons tests were performed with two and greater than two groups, respectively, to compare the tumor growth, in vitro time-course experiments, or in vitro T cell experiments. Log-rank Mantel-Cox test was used for survival analysis. *P* < 0.05 was used as a threshold of statistical significance.

### Supplementary Information

Supplementary Information.

## Data Availability

The data generated in this study are available within the article and its supplementary data files or upon request from the corresponding author.

## References

[1] Sung H (2021). Global cancer statistics 2020: GLOBOCAN estimates of incidence and mortality worldwide for 36 cancers in 185 countries. CA Cancer J. Clin..

[2] Harbeck N (2019). Breast cancer.. Nat. Rev. Dis. Primers.

[3] Cortes J (2020). Pembrolizumab plus chemotherapy versus placebo plus chemotherapy for previously untreated locally recurrent inoperable or metastatic triple-negative breast cancer (KEYNOTE-355): A randomised, placebo-controlled, double-blind, phase 3 clinical trial. Lancet.

[4] Schmid P (2020). Pembrolizumab for early triple-negative breast cancer. N. Engl. J. Med..

[5] FDA. FDA approves pembrolizumab for high-risk early-stage triple-negative breast cancer. 221. https://www.fda.gov/drugs/resources-information-approved-drugs/fda-approves-pembrolizumab-high-risk-early-stage-triple-negative-breast-cancer. Accessed 7 May 2023.

[6] FDA. FDA grants accelerated approval to pembrolizumab for locally recurrent unresectable or metastatic triple negative breast cancer. 2020. https://www.fda.gov/drugs/resources-information-approved-drugs/fda-grants-accelerated-approval-pembrolizumab-locally-recurrent-unresectable-or-metastatic-triple?utm_medium=email&utm_source=govdelivery. Accessed 7 May 2023.

[7] FDA. FDA grants accelerated approval to dostarlimab-gxly for dMMR advanced solid tumors. 2021. https://www.fda.gov/drugs/resources-information-approved-drugs/fda-grants-accelerated-approval-dostarlimab-gxly-dmmr-advanced-solid-tumors. Accessed 7 May 2023.

[8] Solinas C (2017). Targeting immune checkpoints in breast cancer: An update of early results. ESMO Open.

[9] Hanna A, Balko JM (2021). Breast cancer resistance mechanisms: Challenges to immunotherapy. Breast Cancer Res. Treat..

[10] Martins F (2019). Adverse effects of immune-checkpoint inhibitors: Epidemiology, management and surveillance. Nat. Rev. Clin. Oncol..

[11] Karn T (2020). Tumor mutational burden and immune infiltration as independent predictors of response to neoadjuvant immune checkpoint inhibition in early TNBC in GeparNuevo. Ann. Oncol..

[12] Schmid P (2020). Pembrolizumab plus chemotherapy as neoadjuvant treatment of high-risk, early-stage triple-negative breast cancer: Results from the phase 1b open-label, multicohort KEYNOTE-173 study. Ann. Oncol..

[13] Loi S (2019). Tumor-infiltrating lymphocytes and prognosis: A pooled individual patient analysis of early-stage triple-negative breast cancers. J. Clin. Oncol..

[14] Routy B (2018). Gut microbiome influences efficacy of PD-1-based immunotherapy against epithelial tumors. Science.

[15] Gopalakrishnan V (2018). Gut microbiome modulates response to anti-PD-1 immunotherapy in melanoma patients. Science.

[16] Matson V (2018). The commensal microbiome is associated with anti-PD-1 efficacy in metastatic melanoma patients. Science.

[17] Nejman D (2020). The human tumor microbiome is composed of tumor type-specific intracellular bacteria. Science.

[18] Galeano Nino JL (2022). Effect of the intratumoral microbiota on spatial and cellular heterogeneity in cancer. Nature.

[19] Fu A (2022). Tumor-resident intracellular microbiota promotes metastatic colonization in breast cancer. Cell.

[20] Tzeng A (2021). Human breast microbiome correlates with prognostic features and immunological signatures in breast cancer. Genome Med..

[21] Zhang B (2019). Inflammatory activation of microglia by staphylococcus aureus caused phenotypic alterations and affected glioblastoma growth. Cell Biochem. Funct..

[22] Chiappini A (2021). Longer survival of glioblastoma complicated by bacterial infections after surgery: What is known today. J. Neurosurg. Sci..

[23] DeNardo DG, Ruffell B (2019). Macrophages as regulators of tumour immunity and immunotherapy. Nat. Rev. Immunol..

[24] Nguyen TH (2022). Rapid pathogen-specific recruitment of immune effector cells in the skin by secreted toxins. Nat. Microbiol..

[25] Gomez MI (2004). *Staphylococcus aureus* protein a induces airway epithelial inflammatory responses by activating TNFR1. Nat. Med..

[26] Craven RR (2009). *Staphylococcus aureus* alpha-hemolysin activates the NLRP3-inflammasome in human and mouse monocytic cells. PLoS One.

[27] Liu J (2016). Improved efficacy of neoadjuvant compared to adjuvant immunotherapy to eradicate metastatic disease. Cancer Discov..

[28] Kasikara C (2019). Pan-TAM tyrosine kinase inhibitor BMS-777607 Enhances Anti-PD-1 mAb efficacy in a murine model of triple-negative breast cancer. Cancer Res..

[29] Shi J (2015). Cleavage of GSDMD by inflammatory caspases determines pyroptotic cell death. Nature.

[30] Su L (2023). Targeting Src reactivates pyroptosis to reverse chemoresistance in lung and pancreatic cancer models. Sci. Transl. Med..

[31] Wang Y (2017). Chemotherapy drugs induce pyroptosis through caspase-3 cleavage of a gasdermin. Nature.

[32] Lu L (2022). Emerging mechanisms of pyroptosis and its therapeutic strategy in cancer. Cell Death Discov..

[33] Song L (1996). Structure of staphylococcal alpha-hemolysin, a heptameric transmembrane pore. Science.

[34] Bhakdi S, Tranum-Jensen J (1991). Alpha-toxin of staphylococcus aureus. Microbiol. Rev..

[35] Debien V (2023). Immunotherapy in breast cancer: An overview of current strategies and perspectives. NPJ Breast Cancer.

[36] Bianchini G, De Angelis C, Licata L, Gianni L (2022). Treatment landscape of triple-negative breast cancer—Expanded options, evolving needs. Nat. Rev. Clin. Oncol..

[37] Li Y (2021). Targeted immunotherapy for HER2-low breast cancer with 17p loss. Sci. Transl. Med..

[38] Hutchinson KE (2020). Comprehensive profiling of poor-risk paired primary and recurrent triple-negative breast cancers reveals immune phenotype shifts. Clin. Cancer Res..

[39] Zhu L (2019). Metastatic breast cancers have reduced immune cell recruitment but harbor increased macrophages relative to their matched primary tumors. J. Immunother. Cancer.

[40] Park YH (2020). Chemotherapy induces dynamic immune responses in breast cancers that impact treatment outcome. Nat. Commun..

[41] Voorwerk L (2019). Immune induction strategies in metastatic triple-negative breast cancer to enhance the sensitivity to PD-1 blockade: The TONIC trial. Nat. Med..

[42] St Jean AT, Swofford CA, Panteli JT, Brentzel ZJ, Forbes NS (2014). Bacterial delivery of *Staphylococcus aureus* alpha-hemolysin causes regression and necrosis in murine tumors. Mol. Ther..

[43] Swofford CA, St Jean AT, Panteli JT, Brentzel ZJ, Forbes NS (2014). Identification of *Staphylococcus aureus* alpha-hemolysin as a protein drug that is secreted by anticancer bacteria and rapidly kills cancer cells. Biotechnol. Bioeng..

[44] Alizadeh S, Barzegari A, Esmaeili A, Omidi Y (2020). Designing a light-activated recombinant alpha hemolysin for colorectal cancer targeting. Bioimpacts.

[45] Melero I, Castanon E, Alvarez M, Champiat S, Marabelle A (2021). Intratumoural administration and tumour tissue targeting of cancer immunotherapies. Nat. Rev. Clin. Oncol..

[46] Humeau J, Le Naour J, Galluzzi L, Kroemer G, Pol JG (2021). Trial watch: Intratumoral immunotherapy.. Oncoimmunology.

[47] Hong WX (2020). Intratumoral immunotherapy for early-stage solid tumors. Clin. Cancer Res..

[48] Yau C (2022). Residual cancer burden after neoadjuvant chemotherapy and long-term survival outcomes in breast cancer: A multicentre pooled analysis of 5161 patients. Lancet Oncol..

[49] Brummel K, Eerkens AL, de Bruyn M, Nijman HW (2023). Tumour-infiltrating lymphocytes: From prognosis to treatment selection. Br. J. Cancer.

[50] Shi J, Gao W, Shao F (2017). Pyroptosis: Gasdermin-mediated programmed necrotic cell death. Trends Biochem. Sci..

[51] Thompson DA, Weigel RJ (1998). Characterization of a gene that is inversely correlated with estrogen receptor expression (ICERE-1) in breast carcinomas. Eur. J. Biochem..

[52] Zhang Z (2020). Gasdermin E suppresses tumour growth by activating anti-tumour immunity. Nature.

[53] Rogers C (2019). Gasdermin pores permeabilize mitochondria to augment caspase-3 activation during apoptosis and inflammasome activation. Nat. Commun..

[54] Moller N (2020). S. aureus alpha-toxin monomer binding and heptamer formation in host cell membranes—Do they determine sensitivity of airway epithelial cells toward the toxin?. PLoS One.

[55] Wilke GA, Bubeck Wardenburg J (2010). Role of a disintegrin and metalloprotease 10 in staphylococcus aureus alpha-hemolysin-mediated cellular injury. Proc. Natl. Acad. Sci. U. S. A..

[56] Hildebrand A, Pohl M, Bhakdi S (1991). *Staphylococcus aureus* alpha-toxin. Dual mechanism of binding to target cells. J. Biol. Chem..

[57] Cheng Y (2021). ADAM10 is involved in the oncogenic process and chemo-resistance of triple-negative breast cancer via regulating Notch1 signaling pathway, CD44 and PrPc. Cancer Cell. Int..

[58] Mullooly M (2015). ADAM10: A new player in breast cancer progression?. Br. J. Cancer.

[59] Wei Y (2022). Bidirectional functional effects of *Staphylococcus* on carcinogenesis. Microorganisms.

[60] Vandenesch F, Lina G, Henry T (2012). *Staphylococcus aureus* hemolysins, bi-component leukocidins, and cytolytic peptides: A redundant arsenal of membrane-damaging virulence factors?. Front. Cell. Infect. Microbiol..

[61] Shivaee A, Sedighi M, Imani Fooladi AA (2020). *Staphylococcal* enterotoxins as good candidates for cancer immunotherapy: A systematic review. Ann. Ig..

[62] Parida S (2021). A procarcinogenic colon microbe promotes breast tumorigenesis and metastatic progression and concomitantly activates notch and beta-catenin axes. Cancer Discov..

[63] Velicer CM (2004). Antibiotic use in relation to the risk of breast cancer. JAMA.

[64] Friedman GD (2006). Antibiotics and risk of breast cancer: Up to 9 years of follow-up of 2.1 million women. Cancer Epidemiol. Biomark. Prev..

[65] Zhang X (2021). Antibiotics modulate neoadjuvant therapy efficiency in patients with breast cancer: A pilot analysis. Sci. Rep..

[66] Morrell S (2022). Antibiotic exposure within six months before systemic therapy was associated with lower cancer survival. J. Clin. Epidemiol..

[67] Ransohoff JD (2023). Antimicrobial exposure is associated with decreased survival in triple-negative breast cancer. Nat. Commun..

[68] An J, Kwon H, Lim W, Moon BI (2022). *Staphylococcus aureus*-derived extracellular vesicles enhance the efficacy of endocrine therapy in breast cancer cells. J. Clin. Med..

[69] Urbaniak C (2016). The microbiota of breast tissue and its association with breast cancer. Appl. Environ. Microbiol..

[70] Deng W (2022). *Streptococcal* pyrogenic exotoxin B cleaves GSDMA and triggers pyroptosis. Nature.

[71] Bankhead P (2017). QuPath: Open source software for digital pathology image analysis. Sci. Rep..

