# Supplementary material for: Characterization of immunomodulating agents from Staphylococcus aureus for priming immunotherapy in triple-negative breast cancers
Source: Sci Rep. 2024 Jan 8;14:756. doi: 10.1038/s41598-024-51361-8 (PMC10774339; doi:10.1038/s41598-024-51361-8)

Supplementary information

Characterization of immunomodulating agents from *Staphylococcus aureus* for priming immunotherapy in triple-negative breast cancers

Chin-Chih Liu, Matthew Wolf, Ruth Ortego, Dennis Grencewicz, Tammy Sadler, Charis Eng

Supplementary figures and legends 1 to 11

Source data 1 and 2 for immunoblots

Supplementary Figure S1

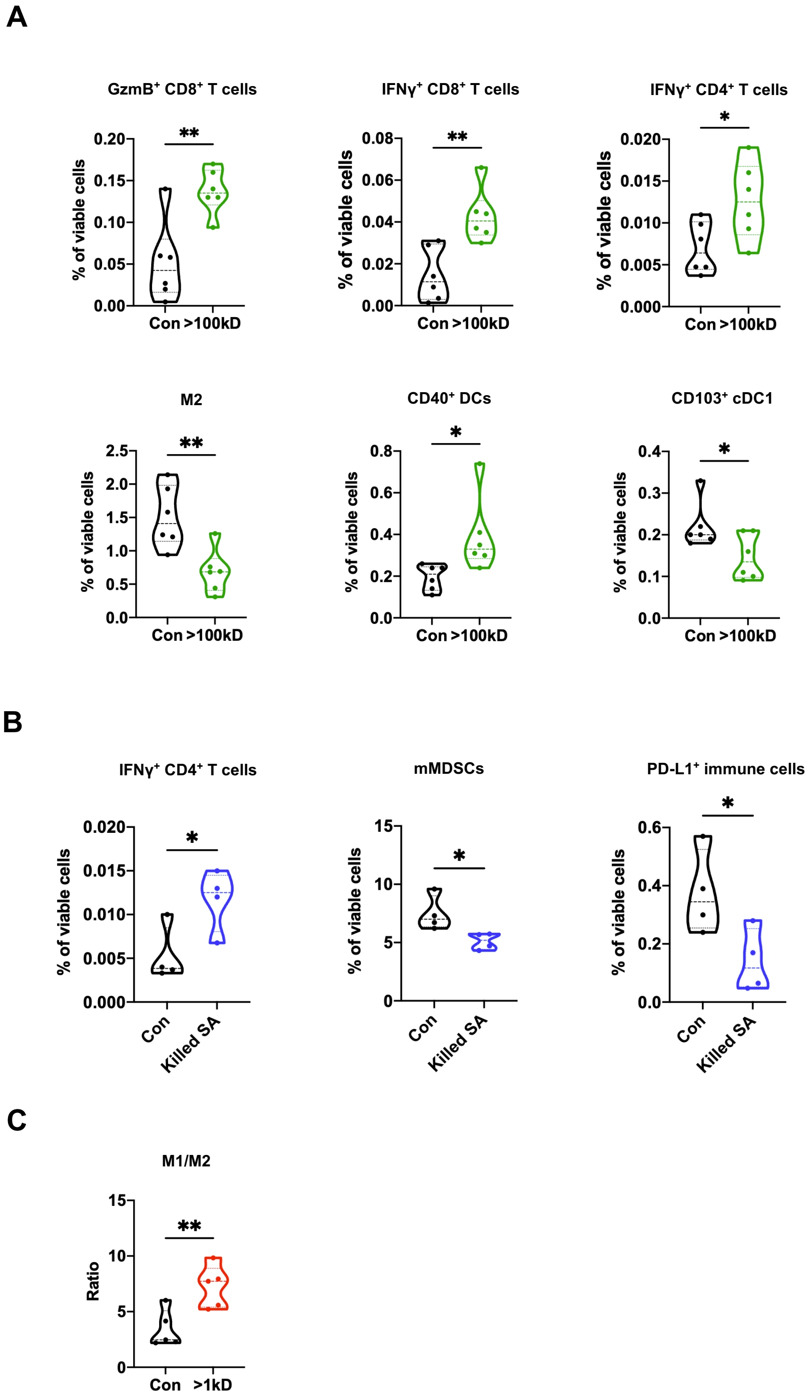

Supplementary Figure S1. Immunomodulating effects of *S. aureus* derivatives on T cell activity and innate immune cells. A, Flow cytometric analysis of GzmB$^+$ and IFNγ$^+$ CD8$^+$ T cells, IFNγ$^+$ CD4$^+$ T cells, M2-like macrophages (M2), CD40$^+$ DCs, and CD103$^+$ conventional type 1 dendritic cells (cDC1) in 4T1 tumors injected with >100 kD molecules from *S. aureus* spent media (>100kD, in green, n=6) or control media (Con, in black, n=6). **B,** Flow cytometric analysis of IFNγ$^+$ CD4$^+$ T cells, monocytic myeloid-derived suppressor cells (mMDSCs), and PD-L1$^+$ CD45$^+$ immune cells in 4T1 tumors injected with heat-killed *S. aureus* (killed SA, in blue, n=4) control DPBS (Con, in black, n=4). **C,** Flow cytometric analysis of M1/M2 in 4T1 tumors injected with >1 kD molecules from *S. aureus* spent media (>1kD, in red, n=5) or control media (Con, in black, n=5). Data are presented as median with quartiles (truncated violin plots). Unpaired two-tailed Student's *t*-test. *, $P < 0.05$; **, $P < 0.01$.

Supplementary Figure S2

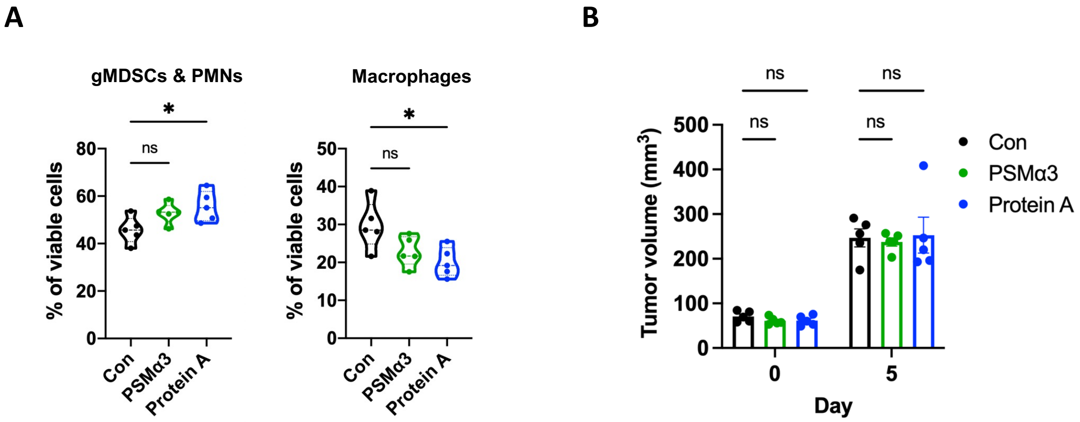

Supplementary Figure S2. Effects of Protein A and PSMα3 treatment on innate immune cells in TIMEs and tumor growth. **A** and **B**, 4T1 tumors were injected with 20 μg of PSMα3 (in green, n=5), 4 μg of Protein A (in blue, n=5), or vehicle control (Con, in black, n=5) on day 0. Flow cytometric analysis of $Ly6C^{hi}Ly6G^{-}CD11b^{+}CD45^{+}$ (gMDSCs and PMNs) and macrophages six days after the injections (**A**). Tumor volumes were gauged on day 0 and day 5 (**B**). Data are presented as median with quartiles (truncated violin plots) (**A**) or mean ± s.e.m (**B**). One-way analysis of variance (ANOVA) (**A**). Two-way analysis of variance (ANOVA) with multiple comparisons (**B**). *, $P < 0.05$; ns, not significant.

Supplementary Figure S3

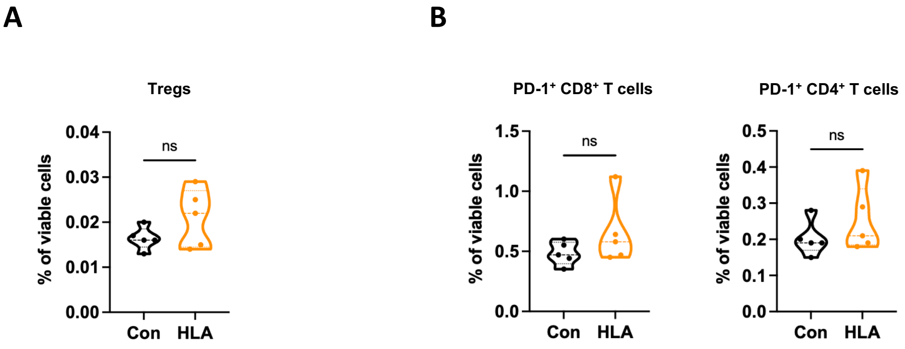

Supplementary Figure S3. α-hemolysin did not affect the abundance of Tregs and PD-1$^+$ T cells in the tumor immune microenvironment. A and **B**, Six days after 4T1 tumors were injected with 1 μg of HLA (in orange, n=5) or vehicle control (in black, n=5), flow cytometric analysis was performed to enumerate the percentage of regulatory T cells (Tregs) (**A**) and PD-1$^+$ CD8$^+$ and PD-1$^+$ CD4$^+$ T cells (**B**) among viable cells. Data are presented as median with quartiles (truncated violin plots). Unpaired two-tailed Student's *t*-test. ns, not significant.

Supplementary Figure S4

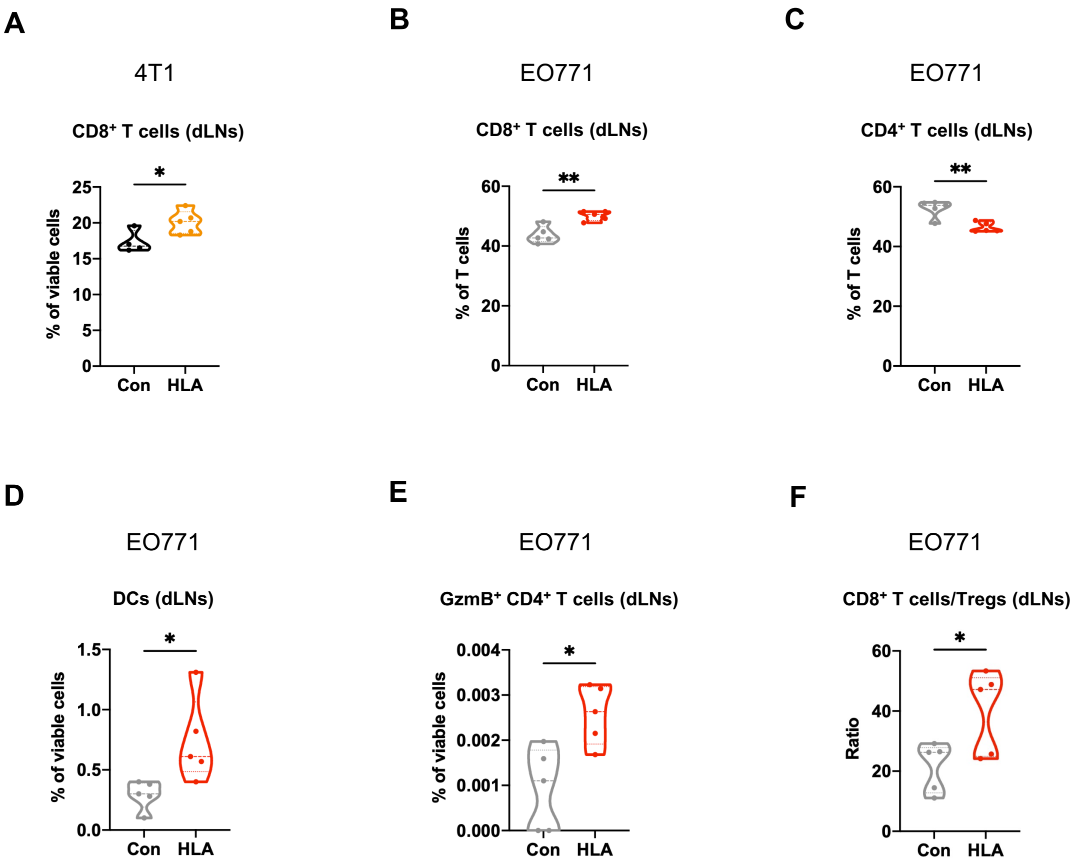

Supplementary Figure S4. Intratumoral HLA injection affects immune cell population in tumor-draining lymph nodes (dLNs). A, Six days after the 4T1 tumors were administered with 1 μg of HLA (in orange, n=4) or vehicle control (Con, in black, n=5), the percentage of $CD8^+$ T cells among viable cells in dLNs was analyzed. **B** and **C,** Six days after the EO771 tumors were administered with 2 μg of HLA (in red, n=5) or vehicle control (Con, in gray, n=5), the percentage of $CD8^+$ (**B**) and $CD4^+$ T cells (**C**) among total T cells in dLNs were gauged. **D-F,** Acute effects of intratumoral HLA injections on dLNs of EO771 tumors. EO771 tumors were injected with 2ug of HLA (in red, n=5) or vehicle control (Con, in gray, n=5) on day 0 and day 5. dLNs were collected on day 6 and flow cytometric analysis was performed to gauge DCs (**D**), $GzmB^+$ $CD4^+$ T cells (**E**), and the ratio of $CD8^+$ T cells to regulatory T cells (Tregs) (**F**). Data are presented as median with quartiles (truncated violin plots). Unpaired two-tailed Student's *t*-test. *, $P < 0.05$; **, $P < 0.01$.

Supplementary Figure S5

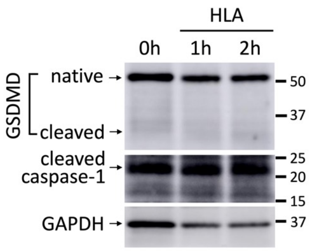

Supplementary Figure S5. HLA did not induce the expression of cleaved GSDMD and cleaved Caspase-1. EO771 cells were treated with 200 μg/ml of HLA for 0-2 hours and the protein expression of cleaved GSDMD and cleaved Caspase-1 were analyzed by Western Blot. Original blots are presented in Supplementary Information Source data 2.

Supplementary Figure S6

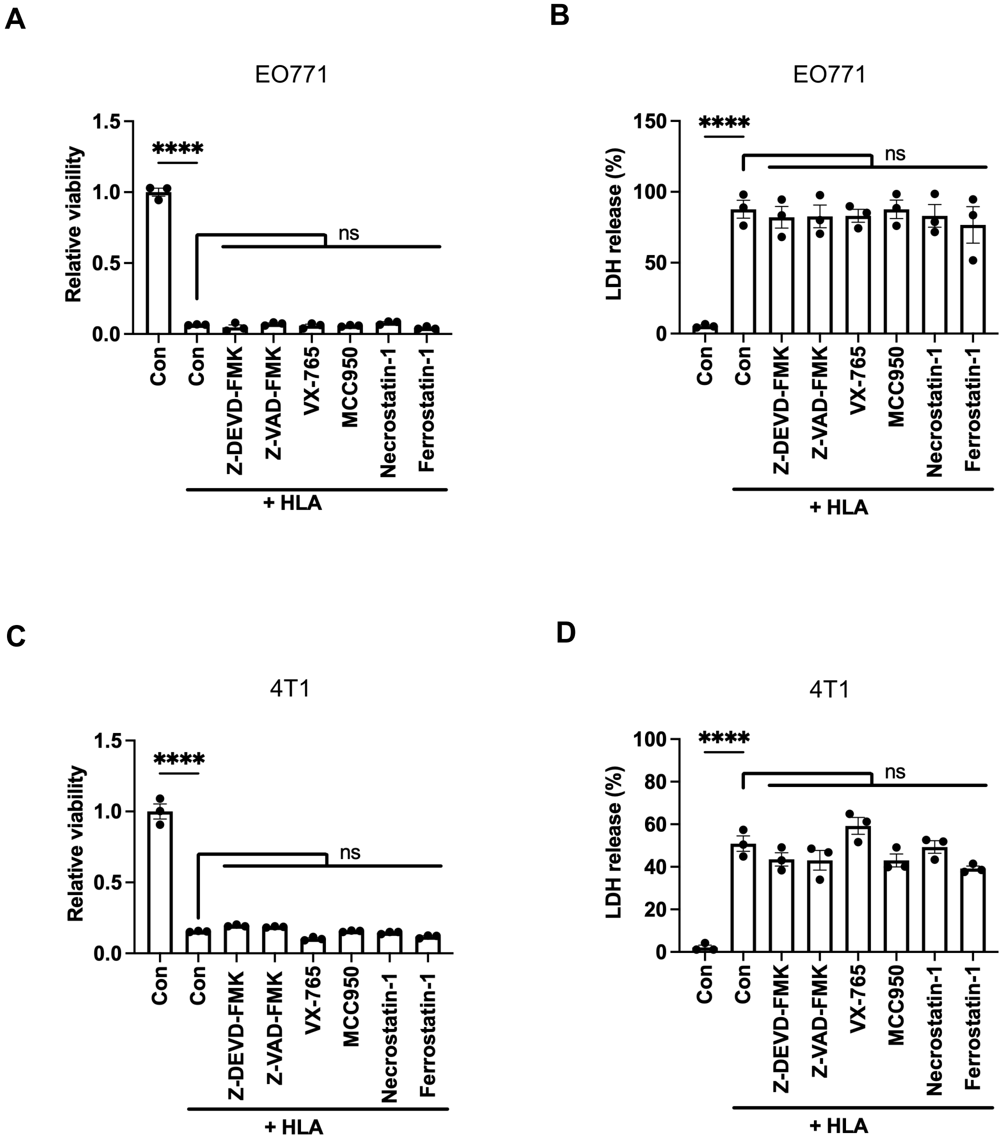

Supplementary Figure S6. HLA-induced cell death is independent of Caspases, NLRP3, necroptosis, and ferroptosis. EO771 (**A** and **B**) and 4T1 cells (**C** and **D**) were pre-treated with the indicated inhibitors and vehicle control (Con) for 1.5 hours, which were followed by the co-treatment of HLA (200 μg/ml for EO771 and 100 μg/ml for 4T1) with the continuous presence of the corresponding inhibitors for 2 hours. The viability (**A** and **C**) and levels of lytic cell death (**B** and **D**) were determined by MTS and LDH release assay, respectively (n=3). Data are presented as mean ± s.e.m. One-way analysis of variance (ANOVA). ****, $P < 0.0001$; ns, not significant.

Supplementary Figure S7

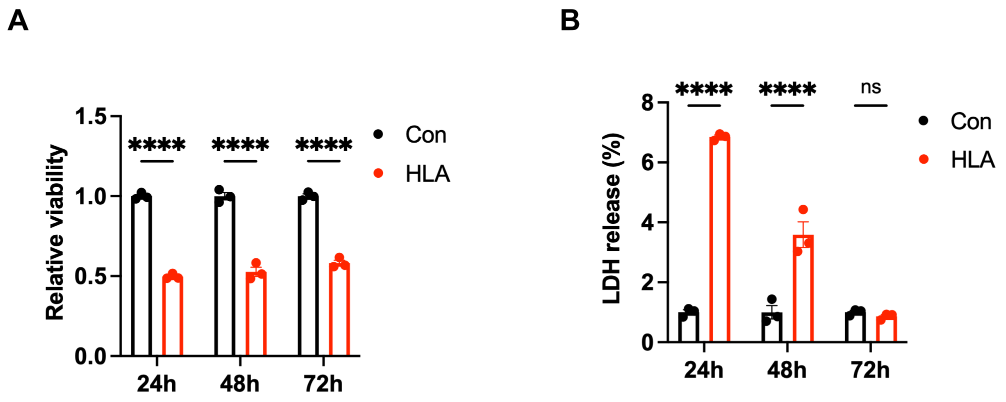

Supplementary Figure S7. The *in vitro* tumoricidal activity of HLA declines after 24 hours of treatment. EO771 cells that were treated with 200 μg/ml HLA for 24 hours, 48 hours, and 72 hours were subjected to the analysis of viability (**A**) and lytic cell death (n=3) (**B**). Data are presented as mean ± s.e.m. Two-way analysis of variance (ANOVA) with multiple comparisons. ****, $P < 0.0001$; ns, not significant.

Supplementary Figure S8

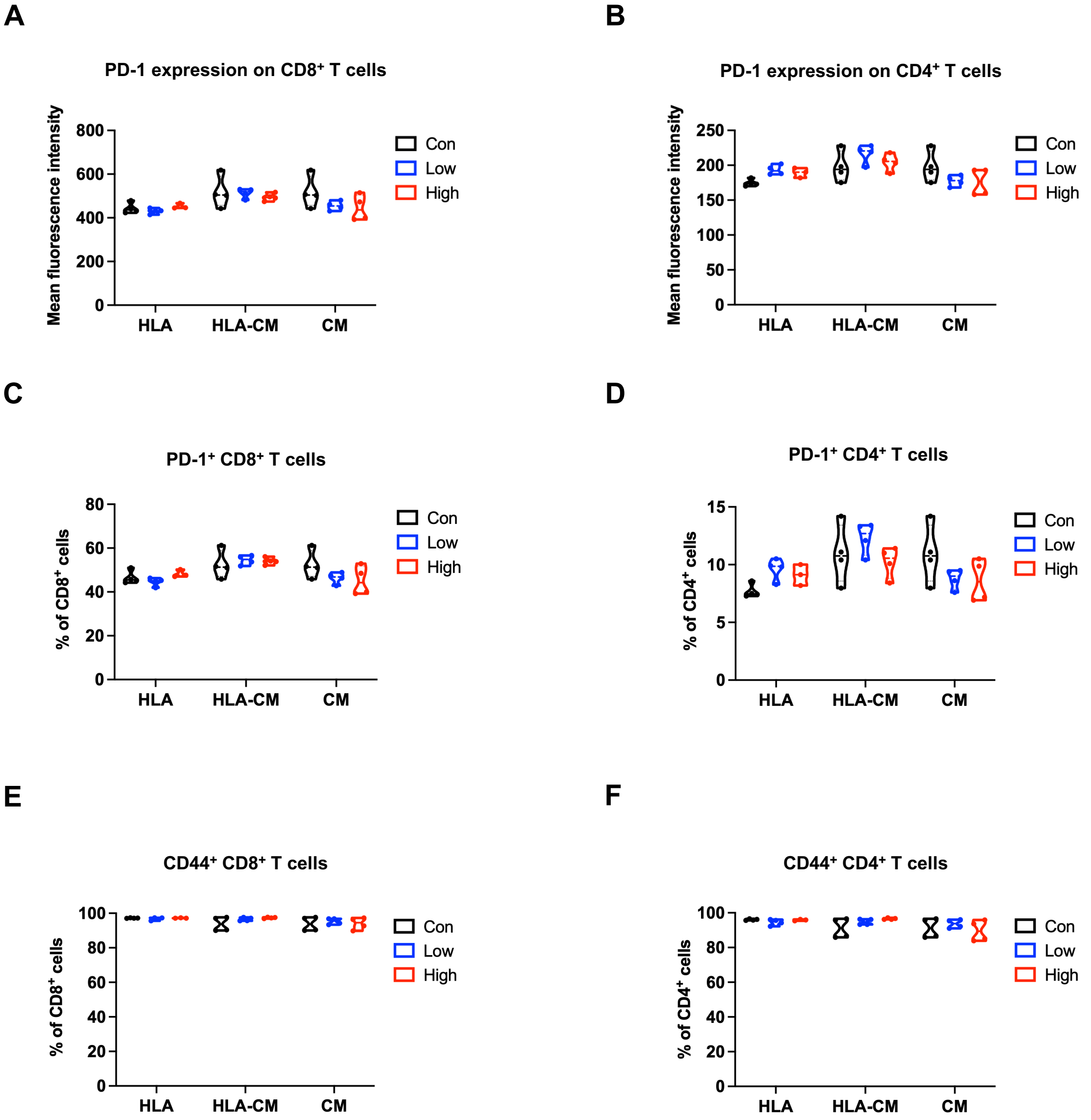

Supplementary Figure S8. HLA treatment did not affect PD-1 expression and activation of T cells *in vitro*. A-F, The *in vitro* expanded splenic T cells were subjected to a 24-hour treatment of either HLA, the conditioned media from the HLA-treated EO771 cells (HLA-CM), or the conditioned media from the vehicle-treated EO771 cells (CM). Details of the treatment conditions refer to Fig. 5 and the Methods. Flow cytometric analysis was performed to determine the expression levels of PD-1 on $CD8^+$ and $CD4^+$ T cells (**A** and **B**), the percentage of PD-1^+ cells among $CD8^+$ and $CD4^+$ populations (**C** and **D**), and the percentage of activated cells ($CD44^+$) among $CD8^+$ and $CD4^+$ populations (E and F). Data are presented as median with quartiles (truncated violin plots). Two-way analysis of variance (ANOVA) with multiple comparisons. No significant differences were identified in this figure.

Supplementary Figure S9

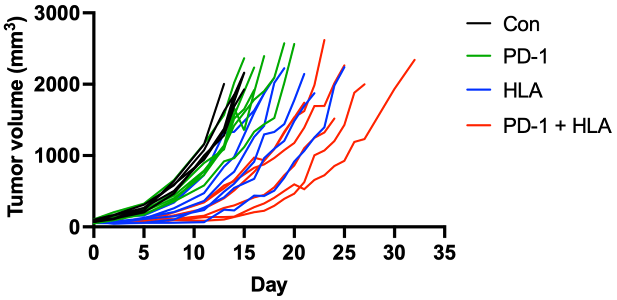

Supplementary Figure S9. The growth curves of individual EO771 tumors that were subjected to different treatments (related to Fig. 6). EO771 tumor-bearing mice were subjected to different treatments with conditions described in Fig. 6. The tumor growth curves of different treatment groups were displayed with different colors. The control group was shown in black (n=5); the HLA group was shown in blue (n=6); the PD-1 group was shown in green (n=6), and the PD-1 + HLA group was shown in red (n=6).

Supplementary Figure S10

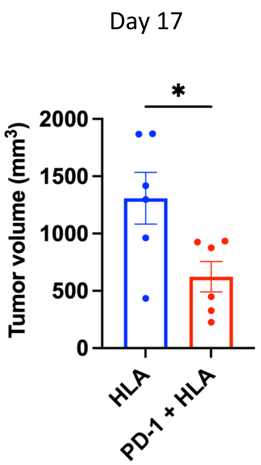

Supplementary Figure S10. Combined treatment of HLA and anti-PD-1 antibody significantly reduces tumor size compared to HLA monotherapy. Comparison of tumor volumes from the mice treated with HLA monotherapy (in blue) and combined treatment of HLA and anti-PD-1 antibody (in red) on the 17th day of treatment. The treatment scheme is shown in **Fig. 6A**. Control treatment and PD-1 monotherapy are not shown here since some of their tumor sizes had reached the end point at this late time point. Data are presented as mean ± s.e.m. Unpaired two-tailed Student's *t*-test. *, $P < 0.05$.

Supplementary Figure S11

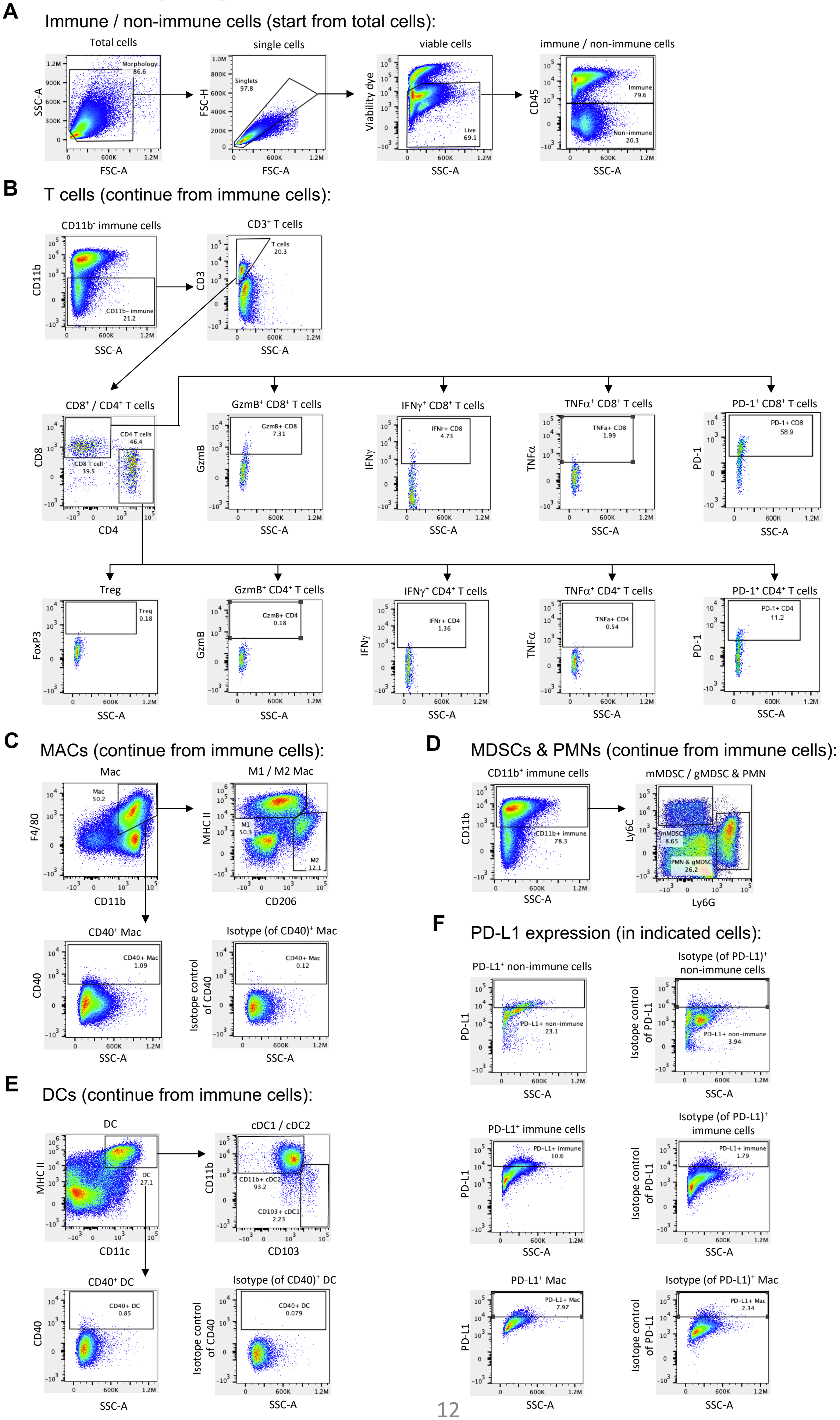

Supplementary Figure S11 (continued)

Supplementary Figure S11. Gating strategy for flow cytometry used throughout manuscript. Gating strategy for immune and non-immune cells in 4T1 tumors (**A**), T cell subsets (**B**), macrophage (MAC) subsets (**C**), mMDSCs and gMDSCs/PMNs (**D**), DC subsets including conventional type 1/2 dendritic cells (cDC1/2) (**E**), PD-L1 expression in immune cells, non-immune cells, and macrophages (MACs) (**F**).

Source data for immunoblots

Source data 1 (of Fig. 5E)

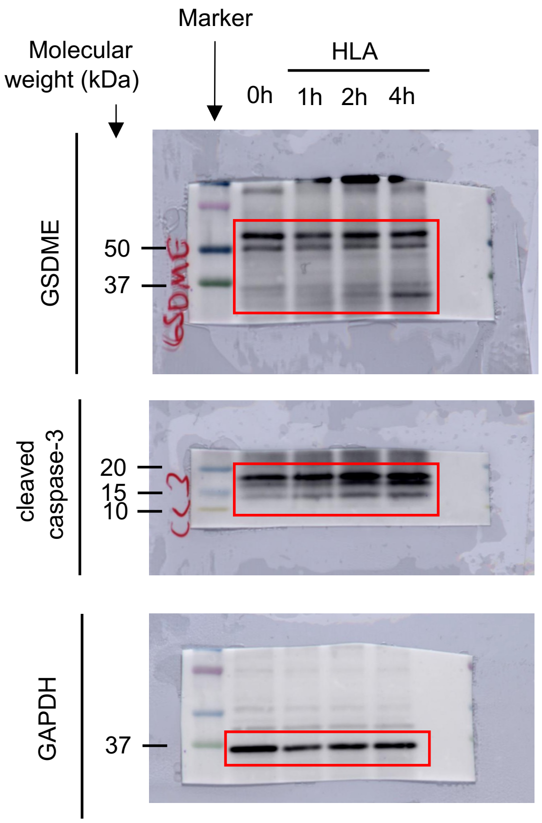

Source data for immunoblots

Source data 2 (of Supplementary Fig. 5)

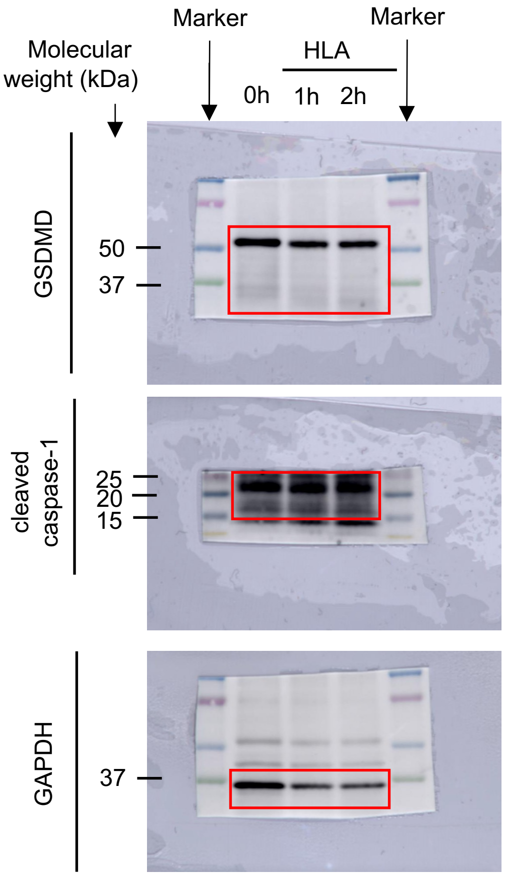

Supplement: Supplementary file 1 — Supplementary Information. [file 41598_2024_51361_MOESM1_ESM.pdf]
